# Microbial changes in stool, saliva, serum, and urine before and after anti-TNF-α therapy in patients with inflammatory bowel diseases

**DOI:** 10.1038/s41598-022-10450-2

**Published:** 2022-04-15

**Authors:** Yong Eun Park, Hye Su Moon, Dongeun Yong, Hochan Seo, Jinho Yang, Tae-Seop Shin, Yoon-Keun Kim, Jin Ran Kim, Yoo Na Lee, Young-Ho Kim, Joo Sung Kim, Jae Hee Cheon

**Affiliations:** 1 Department of Internal Medicine, Yonsei University College of Medicine, Seoul, 03722 Republic of Korea; 2 Division of Gastroenterology, Department of Internal Medicine, Inje University College of Medicine, Haeundae Paik Hospital, Busan, 48108 Republic of Korea; 3 Department of Laboratory Medicine and Research Institute of Bacterial Resistance, Yonsei University College of Medicine, Seoul, 03722 Republic of Korea; 4 MD Healthcare Inc, Seoul, 03923 Republic of Korea; 5 Eisai Korea Inc., Seoul, 06163 Republic of Korea; 6 Samsung Medical Center, Sungkyunkwan University School of Medicine, Seoul, 06351 Republic of Korea; 7 Department of Internal Medicine, Seoul National University College of Medicine, Seoul, 03080 Republic of Korea; 8 Institute of Gastroenterology, Yonsei University College of Medicine, Seoul, 03722 Republic of Korea

**Keywords:** Inflammatory bowel disease, Microbiome

## Abstract

Inflammatory bowel diseases (IBD), including Crohn’s disease and ulcerative colitis, are chronic immune-mediated intestinal inflammatory disorders associated with microbial dysbiosis at multiple sites, particularly the gut. Anti-tumor necrosis factor-α (TNF-α) agents are important treatments for IBD. We investigated whether microbiome changes at multiple sites can predict the effectiveness of such treatment in IBD. Stool, saliva, serum, and urine biosamples were collected from 19 IBD patients before (V1) and 3 months after (V2) anti-TNF-α treatment, and 19 healthy subjects (control). Microbiota analysis was performed using extracellular vesicles (EVs; all four sample types) and next-generation sequencing (NGS; stool and saliva). The stool, using NGS analysis, was the only sample type in which α-diversity differed significantly between the IBD and control groups at V1 and V2. Relative to non-responders, responders to anti-TNF-α treatment had significantly higher levels of *Firmicutes* (phylum), *Clostridia* (class), and *Ruminococcaceae* (family) in V1 stool, and *Prevotella* in V1 saliva. Non-responders had significantly higher V2 serum and urine levels of *Lachnospiraceae* than responders. Finally, *Acidovorax caeni* was detected in all V1 sample types in responders, but was not detected in non-responders. Microbiome changes at multiple sites may predict the effectiveness of anti-TNF-α treatment in IBD, warranting further research.

## Introduction

Inflammatory bowel diseases (IBD) are relapsing chronic inflammatory intestinal disorders that include ulcerative colitis (UC) and Crohn’s disease (CD)^1^. The etiology of IBD is not yet clearly identified, but it is presumed that an abnormal immune response to the damaged mucosal barrier with altered gut microbiota is caused by environmental factors in genetically vulnerable hosts^1,2^. In particular, in patients with IBD, dysbiosis, an imbalance of the gut microbiota that contributes to the host homeostasis is a crucial factor of disease development^1^. Several studies have reported altered compositions of the gut microbiota in IBD patients, with such patients having been shown to have decreased α-diversity of the gut microbiota, lower abundance of *Firmicutes*, and higher abundance of *Proteobacteria*^1,3–5^.

Studies have elucidated the pathogenesis of IBD and developed therapeutic agents for its treatment based on various immunologic and cellular biochemistry mechanisms, but the optimal treatment of IBD based on its pathogenetic mechanisms is not yet clear. Among the current treatment modalities, anti-tumor necrosis factor (TNF) agents are one of the most important therapeutic agents and are now widely used in treating IBD^6^. TNF-α is a proinflammatory cytokine and is produced by activated macrophages, monocytes, and T lymphocytes^7,8^. IBD patients are commonly shown to have increased expression of TNF-α protein and messenger RNA (mRNA)^9^. Therefore, several anti-TNF-α monoclonal antibodies, such as infliximab, adalimumab, golimumab, and certolizumab pegol, have been developed for the treatment of IBD^8,10^. Anti-TNF-α agents have been shown to provide higher rates of remission induction and maintenance, and to induce mucosal healing more frequently, than other conventional treatment modalities^6^. However, up to 30% of IBD patients appear to be primary non-responders who do not clinically benefit from anti-TNF-α induction therapy, while another 30–40% lose response during the first year of the treatment, leading to the need to increase their dosage or switch to another biologic agent^11^. Several reports have shown that drug treatment can improve abnormal fecal microbiota profiles to be similar to normal healthy microbiota, including re-occurrence of *Firmicutes* and *Bacteroidetes*^1,12–14^. However, studies on changes in the microbiota between groups with and without drug treatment are still lacking. In addition, most studies have been conducted using fecal samples, and there are no studies on changes in microbiota in other sites (urine, blood, etc.).

In addition, in recent years, analysis and research on extracellular vesicles (EVs) have gained a lot of attention, and EV analyses in body fluids, as well as in feces, are emerging. Bacterial EVs are nano-sized vesicles in the range of 20–400 nm^15^ made of a lipid bilayer released from cells^16^. EVs contain a variety of biologically active substances, such as proteins, mRNA, microRNA, lipids, and metabolites, that reflect the state of cells^17^, and exist in various body fluids, including blood, urine, saliva, tears, semen, breast milk, and ascites^18^. EVs act as natural messengers involved in cell-to-cell communication within or between host cells and microbial populations, and also act as immune modulators, and virulence and anti-bioresistance factors^19–21^. Therefore, it is possible to extract DNA and analyze microbiota using EVs from various body fluids. However, no studies on microbiota using EV analysis in body fluids such as saliva, urine, and serum have been conducted in IBD patients. Therefore, this study aimed to investigate whether microbiome changes at multiple body sites can predict the effectiveness of anti-TNF-α treatment in IBD patients. Moreover, we sought to find the most suitable sample collection site and biomarker through microbiome analysis at various sites before and after anti-TNF treatment.

## Results

### Baseline patient and control characteristics

Of the 20 healthy subjects who willingly decided to participate in the study, one withdrew consent; therefore, a total of 19 IBD patients and 19 healthy individuals were finally included in the study. The baseline characteristics of the control group, and the IBD group at visit 1 (V1; i.e., before anti-TNF-α therapy) are summarized in Table 1. There were no significant differences between the IBD and control groups with regard to median age (33 vs 31 years; *p* = 0.554) or proportion of males (57.9% vs 68.4%; *p* = 0.735).

**Table 1:** Baseline characteristics of the control and inflammatory bowel disease (IBD) groups before anti-TNF-α therapy (V1), and characteristics of the IBD group 3 months after anti-TNF-α therapy (V2). The independent Student’s t-test (or Mann–Whitney test) was used for continuous variables and the χ^2^ test (or Fisher’s exact test) was used for categorical variables.

Variables	Control (*n* = 19)	IBD group (*n* = 19)		
		V1	V2	*p-*value (V1 vs V2)
Male, **n** (%)	11 (57.9)	13 (68.4)	13 (68.4)	
Age in years, median (IQR)	31 (28–34)	33 (23–52)	33 (23–52)	
**Type of IBD, *n* (%)**				
UC		9 (47.4)	9 (47.4)	
CD		10 (52.6)	10 (52.6)	
**Disease activity, median (IQR)**				
Mayo score [UC patients]		10.0 (9.0–11.5)	3.0 (2.0–5.0)	< 0.001
CDAI [CD patients]		77.2 (45.7–153.0)	40.9 (25.0–54.4)	0.044
**Laboratory findings, median (IQR)**				
ESR, mm/h	< 100	17.5 (2.3–31.8)	12.0 (3.0–24.0)	0.270
C-reactive protein, mg/L		5.2 (2.2–9.0)	0.8 (0.4–3.4)	0.125
Hematocrit, %		40.9 (33.0–45.8)	41.4 (36.8–45.0)	0.374
Fecal calprotectin, μg/g		1572 (748–1800)	378 (145–882)	0.004

*CD* Crohn’s disease, *CDAI* CD activity index, *ESR* erythrocyte sedimentation rate, *IQR* interquartile range, *TNF* tumor necrosis factor, *UC* ulcerative colitis.

The baseline characteristics of CD and UC patients are summarized in Supplementary Table S1. CD patients were significantly younger than UC patients (31 vs 52 years; *p* = 0.021), and more CD than UC patients used an immune-modulator (90.0% vs 33.3%; *p* = 0.011). However, there was no significant difference in sex, underlying diseases, or the use of other medications (Supplementary Table S1).

### Changes in disease activity

After visit 1 (V1), the 19 patients in the IBD group received the following anti-TNF-α agents: infliximab (*n* = 11; 7 with CD and 4 with UC), adalimumab (*n* = 5; 3 with CD and 2 with UC), and golimumab (*n* = 3 with UC). Significant improvements from baseline in disease activity were seen following 3 months of anti-TNF-α treatment, with regard to median Mayo scores in UC patients (10.0 vs 3.0; *p* < 0.001), and median Crohn’s Disease Activity Index (CDAI) score in CD patients (77.2 vs 40.9; *p* = 0.044), as well as median fecal calprotectin levels in all IBD patients (1 572 vs 378 μg/g; *p* = 0.004) (Table 1**)**. However, there was no significant change from baseline in the laboratory findings of erythrocyte sedimentation rate, C-reactive protein, and hematocrit levels at visit 2 (V2; i.e., 3 months after anti-TNF-α treatment) (Table 1**)**.

### Diversity and richness of the microbiota

#### Next-generation sequencing (NGS) analysis in stool and saliva

**Figure 1:**
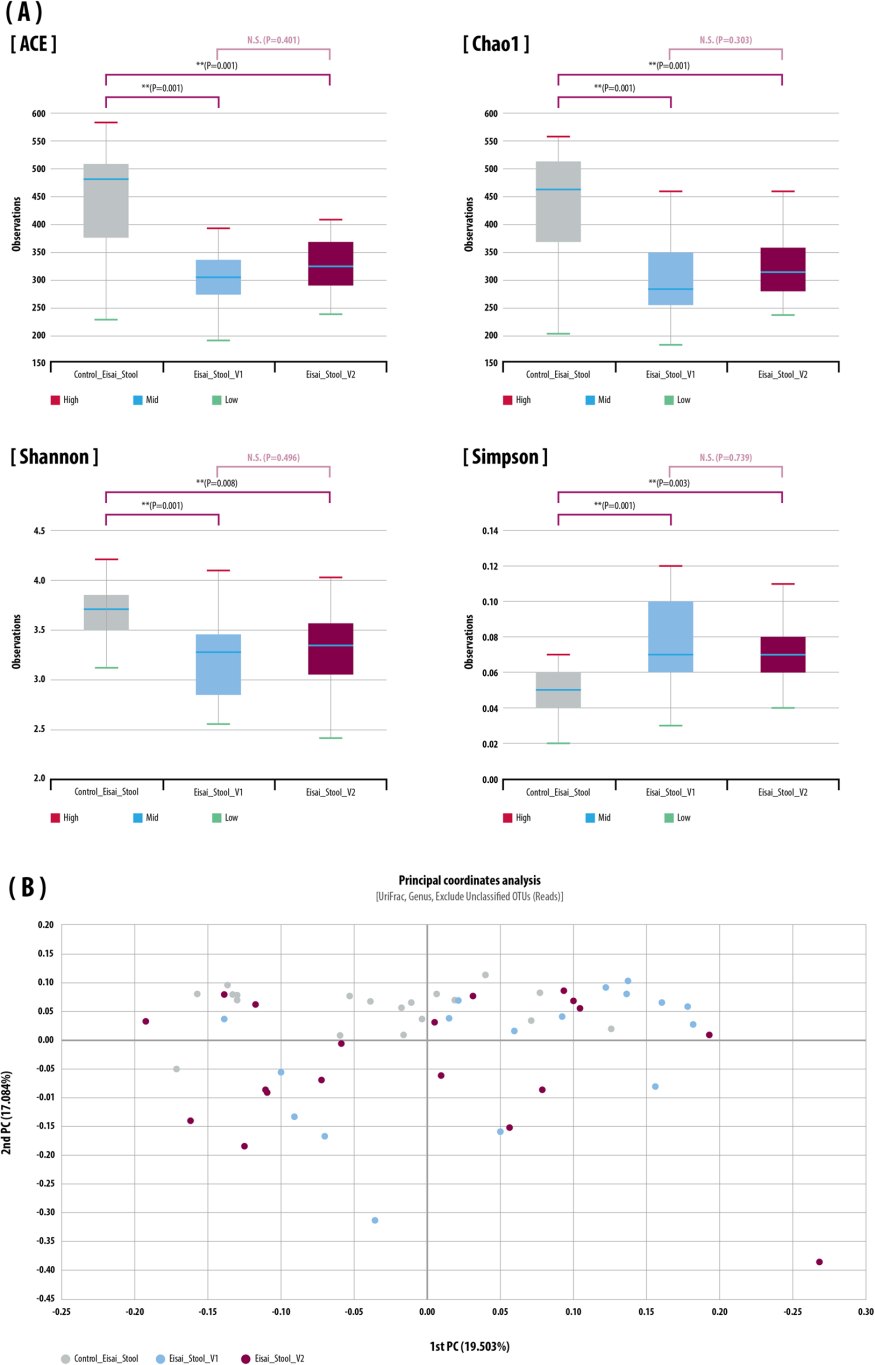
Stool: microbiome diversity based on 16S rRNA gene sequencing in stool in the control and inflammatory bowel disease (IBD) groups before (V1) and after (V2) anti-TNF-α treatment. (**A**) α-Diversity analysis of gut microbiota using ACE, Chao 1, Shannon, and Simpson index. (**B**) β-Diversity analysis of gut microbiota calculated via Principal coordinate analysis (PCoA) scatter plot. Differences between the relative abundance of microbiota were calculated by the Kruskal–Wallis test and Wilcoxon test.

In stool samples, the α-diversity of the control group was significantly greater than that of the IBD patients at V1 and V2 in terms of all types of diversity estimators (ACE, Chao1, Jackknife, Shannon’s diversity index, NP Shannon, and Simpson indexes; all *p* < 0.05) (Fig. 1A and Supplemental Fig. S1A). Regarding β-diversity (Fig. 1B), using the Bray–Curtis dissimilarity and generated principal coordinates analysis (PCoA) plot of the gut microbiota^22,23^, PC 1 was 19.5%, PC 2 was 17.1%, and the IBD group at V2 after anti-TNF- α group was located close to the control group. However, there was no clear clustering, and there was an overlap in gut microbiota in the IBD group at V1 and V2.

In contrast, in the salivary microbiome, there were no significant differences in α-diversity between the control and IBD groups with regard to any of the four types of diversity index (Supplemental Fig. S1B). In addition, there was no significant clustering in the β-diversity of the salivary microbiota between the three groups (PC 1, 29.1%, PC 2, 20.2%) (Supplemental Fig. S1C).

#### EV analyses in stool, saliva, serum, and urine

When ribosomal ribonucleic acid (rRNA) abundance in the microbial EVs from stool, saliva, serum, and urine samples was investigated using a rarefied Chao 1 plot, there was no significant difference in α-diversity between the control group and the IBD group at V1 and V2 (Supplemental Fig. S2). However, with regard to the β-diversity of the stool microbiome, there were significant between-group differences in all of the PCoA plots for phylum, class, order, family, genus, and species (Supplemental Fig. S3).

### Microbiota composition in control and IBD patients

#### NGS analysis in stool and saliva

In 16S rRNA analysis of bulk stool samples, levels of *Actinobacteria* (phylum) and *Ruminococcus* (genus) were higher in the control group than in the IBD patients before anti-TNF-α therapy (V1). After anti-TNF-α therapy (V2), these levels increased to become similar to those of the control group. In contrast, levels of *Enterococcaceae* (family) and *Enterococcus faecium group* (species), which were lower in controls than in IBD patients at V1, significantly decreased to control levels at V2 (all *p* < 0.05) (Supplemental Fig. S4A).

In bulk saliva samples, bacteria levels including *Burkholderiaceae* (family), *Ralstonia* (genus) lower in IBD patients than in the control group at V1, and increased at V2 (Supplemental Fig. S4B).

#### EV analysis in stool, saliva, serum, and urine

In microbial EV analysis of the stool samples, levels of *Proteobacteria* (phylum), *Ruminococcus1, 2* (genus), *Acidovorax caeni* (species), and *Enterococcus faecalis* (species) were higher in the control group than in IBD patients at V1. Conversely, at V1, the IBD group had significantly higher levels of *Veillonella* (genus), *Enterococcus* (genus), and *Clostridiodes difficile* (species) than the control group (Supplemental Fig. S4C).

In microbial EVs from the saliva samples, *Proteobacteria* (phylum), *Burkholderiaceae* (family), *Moraxellaceae* (family), *Acidovorax caeni* (species), and *Enterobacter cloacae* (species) levels were higher in the control group than in IBD patients at V1 **(**Supplemental Fig. S4D).

In microbial EVs from the serum samples, *Proteobacteria* (phylum), *Burkholderiaceae* (family), *Moraxellaceae* (family), *Weeksellaceae* (family), and *Enterobacteriaceae* (family) levels were higher in the control group than in IBD patients at V1. However, *Firmicutes* (phylum), *Clostridia* (class), *Corynebacteriales* (order), and *Ruminococcaceae* (family) levels were lower in the serum of the control group than in IBD patients at V1.

In microbial EVs from the urine samples, *Proteobacteria* (phylum), *Burkholderiaceae* (family), *Moraxellaceae* (family), *Weeksellaceae* (family), and *Enterobacteriaceae* (family) levels were higher in the control group than in IBD patients at V1, whereas levels of *Bacilli* (class) were higher the IBD group.

### Changes in microbiota with anti-TNF treatment

#### Responders versus non-responder

In the NGS analysis of the bulk stool samples, *Actinobacteria* (phylum), *Dorea* (genus), *Agathobaculum* (genus), and *Blautia* (genus) levels were higher at V1 in anti-TNF-α responders than in non-responders. Moreover, *Proteobacteria* (phylum), *Enterobacteriaceae* (family), *Odoribacter* (genus) and *Ruminococcus gnavus* (species) levels were higher in non-responders than in responders at V1 (data not shown).

**Figure 2:**
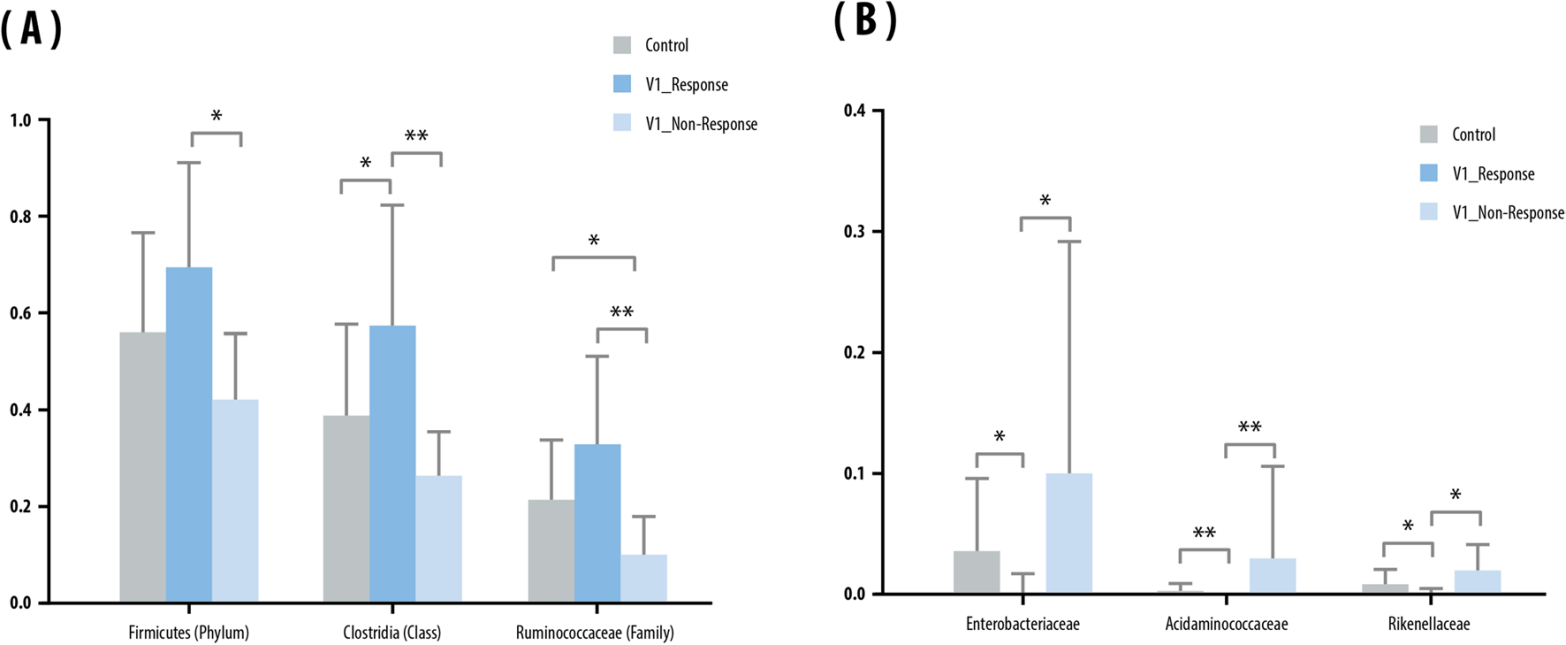
Stool: microbials abundant in the stool of the control and inflammatory bowel disease (IBD) groups before anti-TNF-α treatment (V1). Mean relative abundance of: (**A**) microbials abundant in the responder group (phylum class, family); and (**B**) microbials abundant in the non-responder group (family). Kruskal–Wallis and Wilcoxon tests were used. Bars above columns indicate standard deviation. *p < 0.05; **p < 0.01.

In EV evaluation of differences in microbiota in the stool between IBD patients with or without a treatment response, *Firmicutes* (phylum), *Clostridia* (class), and *Ruminococcaceae* (family) were significantly more abundant at V1 in responders than in non-responders (Fig. 2A). Conversely, non-responders had significantly higher levels of *Enterobacteriaceae*, *Acidaminococcaceae,* and *Rikenellaceae* at the family level than responders at V1 (Fig. 2B).

**Figure 3:**
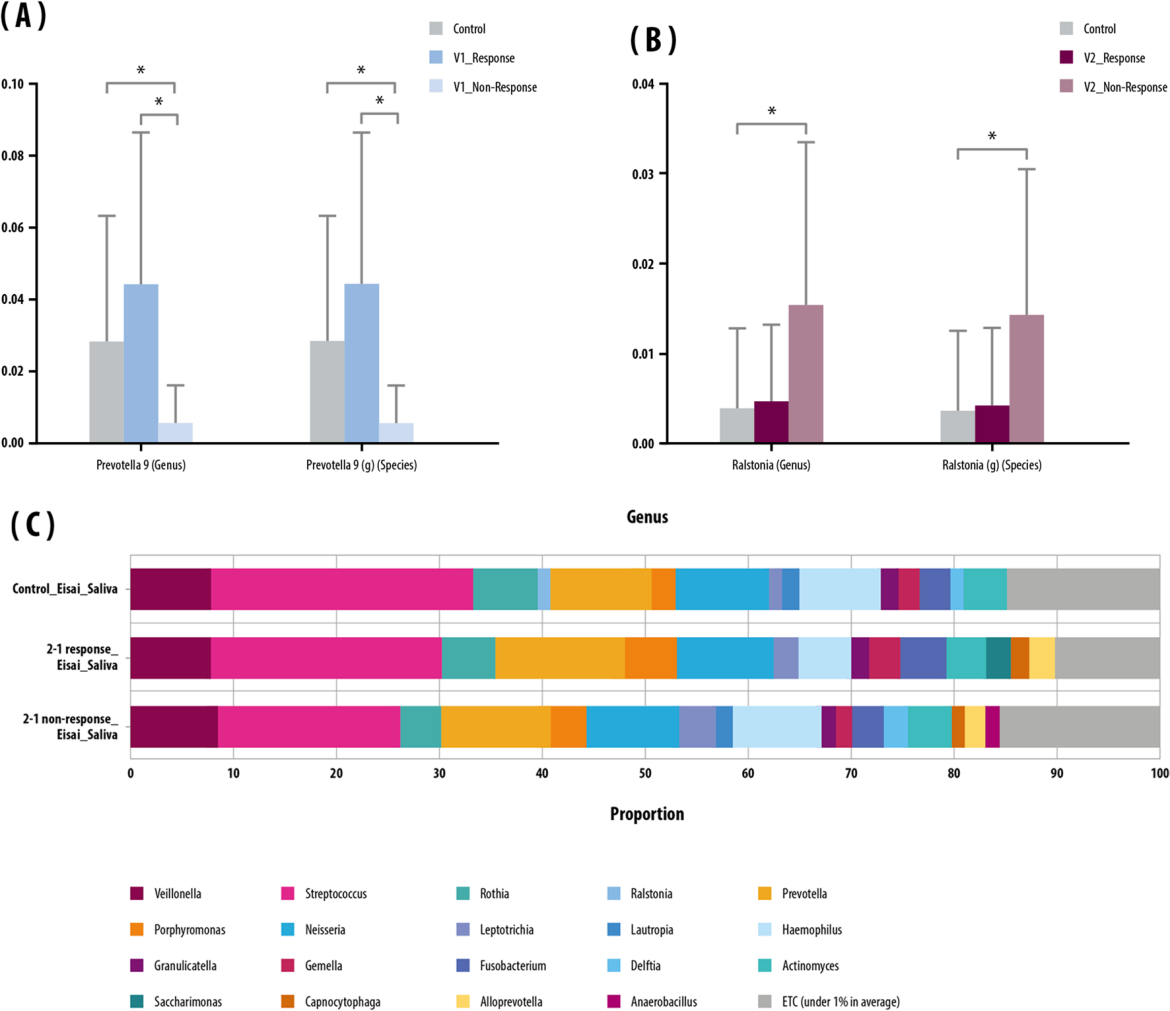
Saliva: microbial composition in the saliva of the control and inflammatory bowel disease (IBD) groups classified by clinical response/non-response. (**A**) Proportion of genera using 16S rRNA. (**B**) Mean relative abundance of genera and species abundant before anti-TNF-α treatment (V1). (**C**) Mean relative abundance of genera and species abundant after anti-TNF-α treatment (V2). Kruskal–Wallis and Wilcoxon tests were used. Bars above columns indicate standard deviation. *p < 0.05.

When NGS analysis was performed on the bulk saliva samples, levels of *Abiotrophia defective*-species, and *FJ976422_s*-species were higher in responders than in non-responders at V1. However, *Ralstonia_f* (family) and *Ralstonia* (genus) levels at V1 were significantly higher in the saliva of non-responders than of responders (Fig. 3A).

In EV analysis of saliva samples in the responder group, the level of *Prevotella 9* in saliva was higher than that of non-responders at V1 (Fig. 3B). In addition, the level of *Ralstonia* was higher at V2 in the non-responder group than in the responder group (Fig. 3C).

**Figure 4:**
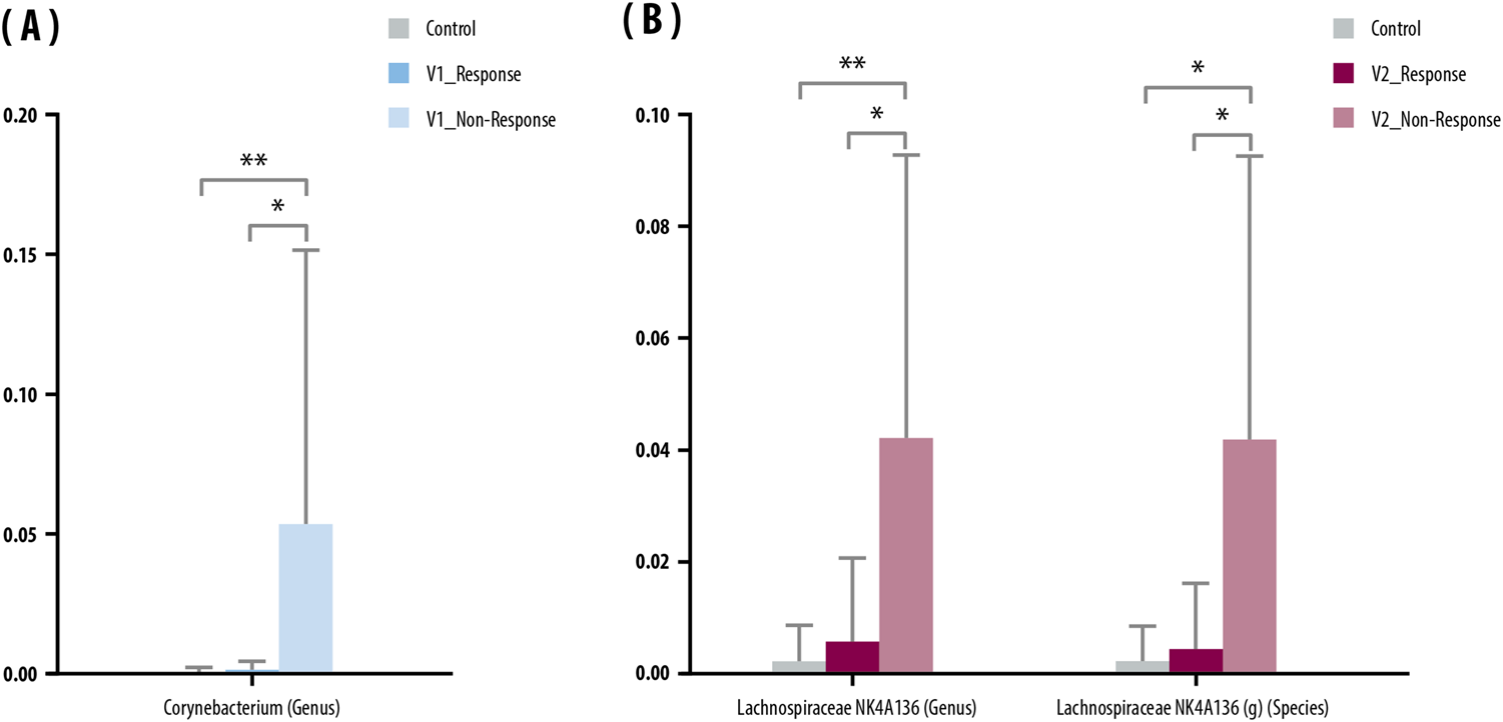
Serum: microbials (genus and/or species) abundant in the serum of the control and inflammatory bowel disease (IBD) groups. Mean relative abundance of: (**A**) microbials abundant in the responder group before anti-TNF-α treatment (V1); and (**B**) microbials abundant in the non-responder group after anti-TNF-α treatment (V2). Kruskal–Wallis and Wilcoxon tests were used. Bars above columns indicate standard deviation. *p < 0.05; **p < 0.01.

In EV analysis of serum samples, *Corynebacterium* was significantly more abundant in non-responders than in responders at V1 (Fig. 4A), and *Lachnospiraceae* was significantly more abundant in non-responders than responders at V2 (Fig. 4B).

**Figure 5:**
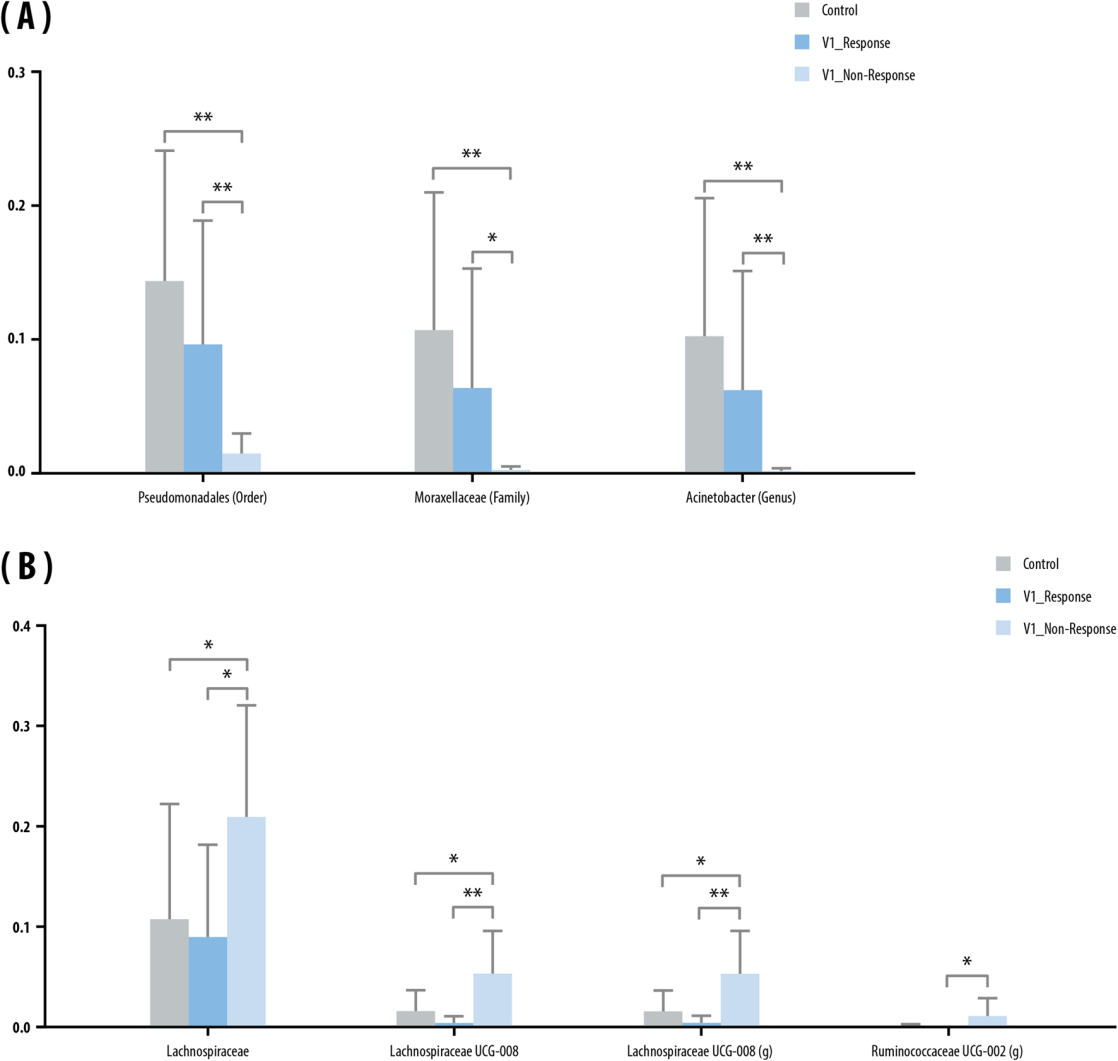
Urine: microbials abundant in the urine of the control and inflammatory bowel disease (IBD) groups before anti-TNF-α treatment (V1). Mean relative abundance of: (**A**) microbials abundant in the responder group; and (**B**) microbials abundant in the non-responder group. Kruskal–Wallis and Wilcoxon tests were used. Bars above columns indicate standard deviation. *p < 0.05; **p < 0.01.

In EV analysis of urine samples, the levels of *Pseudomonadales* (Order), *Moraxellaceae* (Family), and *Acinetobacter* (genus) were significantly higher in responders than in non-responders at V1 (Fig. 5A). However, non-responders had higher levels of *Lachnospiraceae* and *Ruminococcaceae* than responders at V1 (Fig. 5B).

#### Remission versus non-remission

When comparing the microbial EVs of patients who showed remission after anti-TNF-α treatment with those who did not, the results were similar to those of responders versus non-responders. Relative to the remission group at V1, the non-remission group had higher levels of *Clostridia* (Class) in microbial EVs from stool (Supplemental Fig. S5A), and *Corynebacterium* (genus) in microbial EVs from serum (Supplemental Fig. S5B).

#### Prediction of anti-TNF-α response

When the microbiota was analyzed using EVs, *Acidovorax caeni* (species) was found in all types of samples (stool, saliva, serum, and urine) at V1 in responders, as well as in the control group, but not in non-responders (Supplemental Fig. S6A). Likewise, *Acidovorax caeni* was found in all four sample types at V1 in the remission group, but not in the non-remission group (Supplemental Fig. S6B).

Additionally, when examining the *Firmicutes/Bacteroidetes* (F/B) ratio, the microbial EVs from stool F/B ratio was relatively low in the control group, whereas, it was high before anti-TNF-α treatment in the IBD group, and decreased after treatment. Conversely, the F/B ratio in microbial EVs from urine was relatively high in the control group, but was low in the IBD group before treatment, and then increased after treatment. Microbial EVs from saliva F/B ratios were comparable between the control and IBD group at V1, but increased in non-responders at V2. However, there were no significant differences between responders and non-responders with regard to stool, saliva, and urine F/B ratios at either V1 or V2. In microbial EVs from serum, the F/B ratio was significantly higher in responders than in non-responders at V1, but not at V2 (Supplementary Table S2).

### Microbiota composition based on disease activity

**Figure 6:**
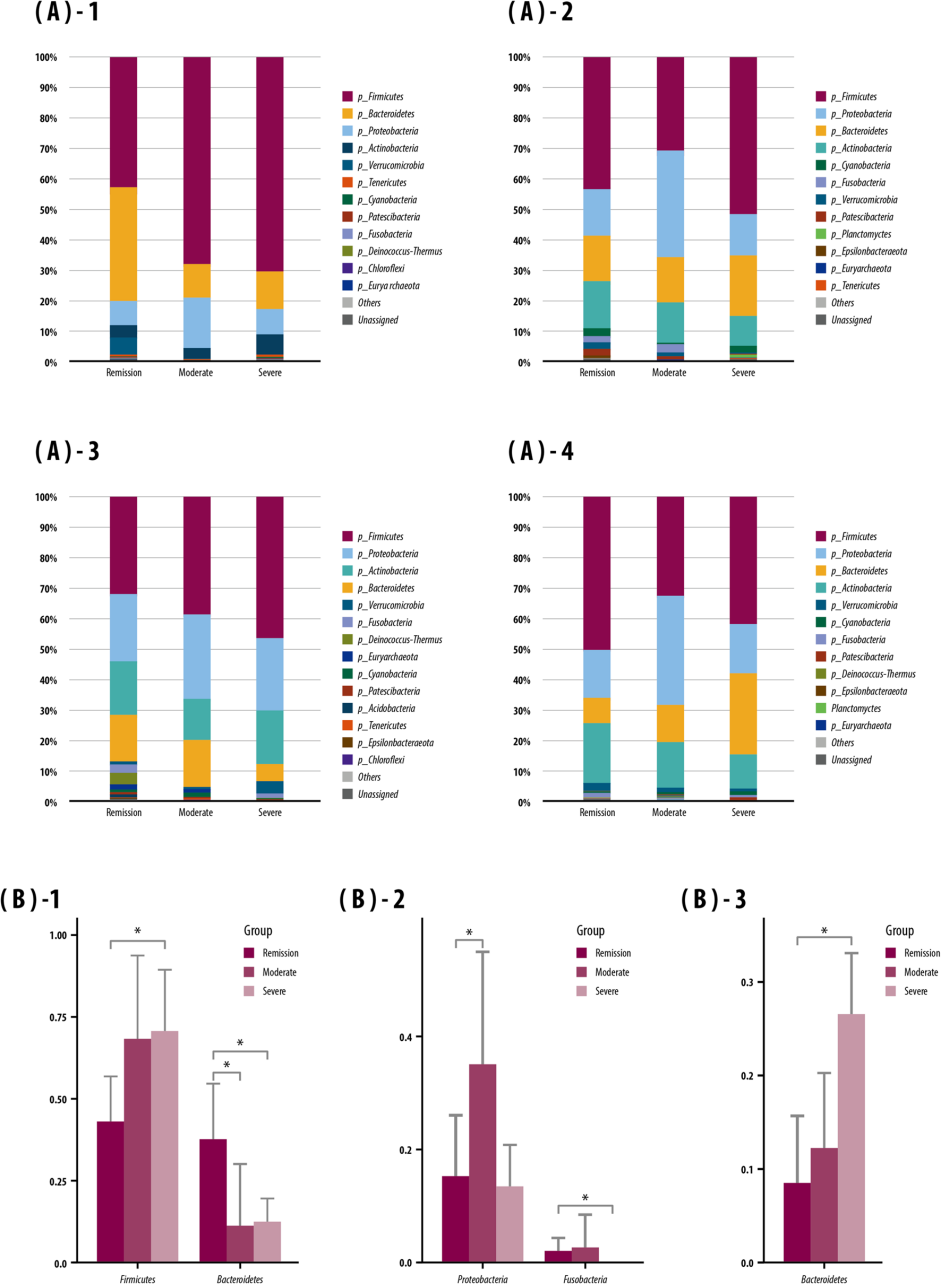
Microbiome composition and microbials showing significant between-group differences in abundance based on baseline disease activity [remission, mild-moderate (labeled as ‘moderate’), or severe] at the phylum level. (**A**) Composition of the microbiome in: (**A-1**) stool; (**A-2**) saliva; (**A-3**) serum; and (**A-4**) urine. (**B**) Microbials showing significant between-group differences in mean relative abundance in: (**B-1**) stool; (**B-2**) saliva; and (**B-3**) urine. Kruskal–Wallis and Wilcoxon tests were used. Bars above columns indicate standard deviation. *p < 0.05.

The effects of disease activity (remission, mild-moderate, or severe) on the microbial EVs at the phylum level in IBD patients were evaluated (Fig. 6). In microbial EVs from stool samples in patients with severe disease activity, the level of *Firmicutes* was significantly higher than that in patients in remission, and that of *Bacteroidetes* was significantly lower than those in patients in remission or mild-moderate disease activity (Fig. 6B-1). In microbial EVs from saliva, *Proteobacteria* and *Fusobacteria* levels were significantly higher in the mild-moderate disease activity group than in the remission group, but did not differ to a significant extent between the severe disease activity and remission groups (Fig. 6B-2). There were no significant differences between the disease severity groups in microbiota at the phylum level in the microbial EVs from serum samples. In microbial EVs from urine samples, *Bacteroidetes* was significantly higher in abundance in patients with severe disease activity than in those in remission. (Fig. 6B-3).

## Discussion

Since the development of anti-TNF-α therapy, the treatment of IBD patients has progressed considerably. Although the treatment needs are not yet fully met, it is still an important treatment modality. In IBD patients, the microbiota plays an important pathogenic role and is a factor that regulates inflammation^24^. It is predicted that the therapeutic efficacy of anti-TNF-α agents may be related to the microbiome not only in the intestines, but also in other body sites, but studies are still lacking. In our study, we examined the differences in the microbiome in stool, saliva, serum, and urine samples using EVs, and investigated the microbiota by comparing taxa in stool and saliva samples using conventional NGS.

Our study was the first to analyze microbiome in various body fluids, such as stool, saliva, serum, and urine, using conventional NGS analysis and nano-particles using EV. Relative to EV analysis, NGS analysis of fecal samples showed a significant reduction in microbiome diversity in IBD patients compared to healthy controls. Although EV analysis did not show any significant difference in terms of microbiome diversity, it showed similar results as the NGS analysis with regard to significant bacterial changes in stool and saliva samples. Analysis of NGS in feces showed lower levels of *Actinobacteria*, *Ruminococcus,* and increased levels of *Enterococcaceae*/*Enterococcus* in IBD patients than in healthy controls, whereas EV analysis showed a decrease in *Proteobacteria*, *Ruminococcus*, *Enterococcaceae/Enterococcus* levels, and an increase in *Clostridiodes difficile* levels in IBD patients. In some cases, such as *Enterococcus*, the results of the NGS and EV analyses were opposite, but the most important results were similar. Differences exist between the NGS and EV analyses, suggesting that EV itself may play a role related to inflammation. However, further research is needed.

EV analysis provided similar results when conducted in saliva, serum, and urine samples in the control and IBD patients. In IBD patients relative to the control group, *Actinobacteria* and *Fusobacteria* were more abundant in saliva samples, *Firmicutes*, *Actinobacteria*, and *Fusobacteria* were more abundant in serum samples, and *Firmicutes* and *Actinobacteria* were more abundant in urine samples. In addition, saliva, serum, and urine samples in the control group showed increased levels of *Proteobacteria* and *Bacteroidetes* compared to those in IBD patients. It appears that the microbiome of feces and the microbiome of saliva, serum, and urine show some opposite tendencies with regard to these bacteria.

We showed that responders to anti-TNF-α treatment had more abundant levels of *Firmicutes* in their stool, and *Prevotella* in their saliva before treatment than non-responders to treatment. In addition, in serum and urine samples, in which the microbiome results of IBD patients were analyzed for the first time, the level of *Lachnospiraceae* was found to be higher in the non-responder group than in the responder group after anti-TNF-α treatment. Furthermore, *Acidovorax caeni* was not detected in the stool, saliva, serum, and urine samples of non-responders before anti-TNF-α treatment (V1), but was found in all samples of responders and control.

The association of *Firmicutes* with IBD is well known. The reduced diversity of gut microbiota in patients with IBD is related to decreased levels of *Firmicutes,* such as the *Clostridium leptum* group and *Faecalibacterium prausnitzii*^1^. *Firmicutes*, especially *F. prausnitzii*, has anti-inflammatory effects by producing substances such as butyrate that can inhibit Th17 cells in IBD^25^. Studies have shown that changes in *Firmicutes* level play a role as an important marker, even during anti-TNF-α treatment. Busquets et al.^14^ reported that the use of adalimumab in CD patients leads to the recovery of *Firmicutes*, *Bacteroides*, and *Actinobacteria*. In addition, Magnusson et al.^26^ reported that patients with CD who were *F. prausnitzii*-rich at baseline responded to anti-TNF-α treatment. Another study showed an increase in the levels of *Lachnospiraceae* and *Blautia* in response to infliximab in CD patients^27^. In addition, *Clostridia* was more abundant in IBD patients who responded to infliximab^27^, and Zhou et al. also reported a higher abundance of *Clostridia* in CD patients who responded to treatment and predicted infliximab effectiveness when combined with fecal calprotectin levels and CDAI^28^. Similar to these previous studies, our study also showed an increase in *Firmicutes* levels in V1 stool of IBD patients who responded to an anti-TNF-α agent. *Firmicutes* levels in feces can be used as a predictive marker for anti-TNF-α treatment effectiveness in IBD.

Until recently, most microbiome research has been focused on the gut, however, new studies are investigating the non-invasive and accessible saliva microbiome. Recent studies have shown that intestinal inflammation and IBD pathogenesis are related by the oral-gut axis connection, in that oral-derived biopathogens translocate to the intestine and cause IBD^29–31^. The major components of the saliva microbiome are *Actinobacteria*, *Bacteroidetes*, *Firmicutes*, *Fusobacteria*, and *Proteobacteria*^32^. Compared to the gut microbiome, the phylum level in saliva shows a similar composition, but it has been reported that there is a difference in the order of abundance^32,33^. Said et al. reported that there was no difference in diversity of salivary microbiota between IBD patients and healthy subjects, with the dominant genera being *Streptococcus*, *Prevotella*, and *Neisseria* in IBD patients^34^. This is similar to the results of this study, in which there was no difference in α-diversity between IBD patients and the healthy control group, showing that in IBD patients, the salivary microbiota may not differ as much as that of the stool. Therefore, it would be difficult to use the change in saliva diversity as a follow-up test. However, our study showed that *Prevotella* was abundantly present at baseline in patients responding to anti-TNF-α treatment, which could be an important factor in patients with IBD. *Prevotella* is a genus of Gram-negative, obligate anaerobe^35^, and reported to be associated with opportunistic infections such as vaginosis, esophagitis, and antral gastritis^36–38^, while most of them are intestinal commensal bacteria in the gut^39^. *Prevotella* can play a role in patients with IBD, as it is reported that it can induce the circulation of bacteria and other inflammatory mediators by inducing inflammation of the mucosa^40^. So far, studies on the saliva microbiome are insufficient, and the association with gut microbiota and in patients with IBD has not yet been clearly identified. In addition, the status of conditions such as tooth decay and periodontitis^41,42^ and diet-related lifestyle can change the composition of the oral microbiome^43^. Although more research is needed, salivary bacteria, such as *Prevotella,* could be used as predictive markers for treatment.

To the best of our knowledge, this is the first study to analyze the microbiome in the serum and urine of IBD patients. Some studies have reported on metabolic interactions in patients with IBD in serum and urine^44,45^. Kolho et al. reported the serum metabolomics in pediatric IBD patients and showed changes in the serum pathways associated with the inflammatory response^44^. However, no previous study has analyzed the microbiome by extracting 16S rRNA from serum and urine. In this study, the non-responder group showed a tendency for higher levels of *Lachnospiraceae* in saliva, serum, and urine samples than the responder and control groups, but the stool samples showed an increase in *Lachnospiraceae* in the control and responders, indicating that the microbiome of saliva/serum/urine and feces showed the opposite tendency. In addition, the level of *Corynebacterium* before treatment was higher in the serum of the non-responder group than in the control and responder groups. *Corynebacterium* is a genus of Gram-positive, aerobic bacteria, with Dinakaran et al.^40^ reporting an increase in these bacteria in colon specimens from patients with IBD. Therefore, *Lachnospiraceae* and *Corynebacterium* have potential as predictive serum markers for treatment response. In urine samples, *Pseudomonadales* (order), *Moraxellaceae* (family), and *Acinetobacter* (genus) belonging to the *Proteobacteria* taxa were higher in abundance before treatment in responders than in non-responders to anti-TNF-α therapy.

In this study, levels of *Proteobacteria* in saliva were higher in the control group than in IBD patients before treatment, and decreased further in IBD patients after treatment. This differs from the results of previous studies^1,28^, which found higher *Proteobacteria* levels in IBD patients than in control, but also showed a significant decrease after treatment, indicating that the treatment effect and *Proteobacteria* levels were related. In addition, in stool samples, patients responding to treatment had lower pre-treatment *Proteobacteria* levels than non-responders. This is also the opposite result of an increase in the baseline *Proteobacteria* levels in the urine of the responder group. These findings seem to show an inverse correlation between the microbial community of feces and serum/urine.

This study showed that *Acidovorax caeni* was observed in the baseline analysis of the responder group, but not in the non-responder group in each of the stool, saliva, serum, and urine samples using EVs. *Acidovorax caeni* is a species of Gram-negative, aerobic bacteria with a polar flagellum^46^; its phylum level is *Proteobacteria*, and it consists of following taxa: *Gammaproteobacteria* (class), *Betaproteobacteriales* (order), *Burkholderiaceae* (family), *Acidovorax* (genus). Increased levels of *Enterobacteriaceae* in IBD patients are well known^1,47,48^, with Alam et al. also showing increased abundance of *Burkholderiaceae* in both CD and UC patients^49^. It is not yet clear whether an increase in *Acidovorax caeni* levels is associated with an increase in *Burkholderiaceae* levels and the potential role that it plays in patients with IBD. It may be a species that predicts the therapeutic effect in patients with IBD because all sample types showed the same results. Further research is needed.

This is the first study in which 16S rRNA was extracted with nano-particles to analyze the microbiome of stool, saliva, serum, and urine in IBD patients. The microbiome in feces and saliva were also analyzed and compared with NGS, and the results were similar to those of previous studies. This showed that it was possible to easily analyze the microbiome, even in other fluids, using EVs However, our study has several limitations. First, a study involving only anti-TNF-α naive patients may result in selection bias. Differences depending on the type and duration of previously used drugs or differences in disease prevalence may affect the results. However, since most of the patients used anti-TNF-α according to the clinical practice guidelines, it is believed that this should not affect the results. Secondly, the analysis was performed by two methods, EVs and NGS. The results of the two methods were not completely identical, which may have the disadvantage of causing potential bias. However, the important results were similar between the two analyses. Intriguingly, some strains were meaningful in EVs, but not in NGS. However, even when both of these analyzes were applied, it was revealed that the strains showing the same results are important in the microbiome of actual IBD patients. Third, due to the fact that the number of subjects in our study was very small and because CD and UC could not be analyzed separately, and only patients from the Seoul area were included, regional differences and selection bias may occur. However, the three university hospitals participating in this study are the largest IBD clinics in Korea and are visited by a large number of IBD patients. However, further studies comparing differences between countries and races are needed. Lastly, there has been no study of the microbiome in serum and urine in IBD, so the implications of the results of this study are yet to be determined. Microbiome studies in saliva are lacking thus far, so studies are needed in the future to further elucidate the role of this microbiome in IBD discovered in this study.

In summary, this study showed that the levels of *Firmicutes* (phylum), *Clostridia* (class), and *Ruminococcaceae* (family) were increased in stool, and the levels of *Prevotella* were increased in saliva at baseline in patients who responded to anti-TNF-α therapy. In serum and urine, the levels of *Lachnospiraceae* were increased in patients in the non-responder group. Finally, *Acidovorax caeni* was found, in all four sample types, only in those IBD patients who responded to anti-TNF-α treatment, so levels of this species may prove helpful in predicting the anti-TNF-α treatment response in IBD patients.

## Materials and methods

### Patients

Between August 2017 and January 2020, we prospectively enrolled 19 patients with IBD and 20 healthy controls at three University Medical Centers, Seoul, Korea. The diagnosis of UC and CD was based on the clinical, endoscopic, histopathologic, and radiologic findings^50–52^. To be included in the IBD group, patients must have met the following criteria: (i) age ≥ 19 years; (ii) had not received antibiotics during the last 3 months; (iii) had not taken probiotics during the last 3 months; (iv) had not previously received anti-TNF-α treatment (i.e., were anti-TNF-α naïve). In addition, the following IBD patients were excluded: (i) women with suspected pregnancy or who were lactating; (ii) patients with conditions that are contraindicated for anti-TNF-α administration, such as the presence of active tuberculosis or other severe infections, such as sepsis or opportunistic infections; (iii) those with no available clinical data such as disease activity or clinical records; and (iv) those who could not be followed up during the study period. To be included in the healthy control group, they must have met the following criteria (i) age ≥ 19 years; (ii) had not received antibiotics during the last 3 months; (iii) had not taken probiotics during the last 3 months; (iv) did not have any intestinal diseases (i.e., irritable bowel syndrome, diverticulitis, microscopic colitis, infective colitis, etc.,).

The baseline characteristics of the patients and healthy individuals were prospectively obtained from the electronic medical data collected, including study subject, disease demographics, comorbid diseases, medication records, and vital signs.

### Ethic declarations

This study was performed in accordance with the ethical guidelines of the 1975 Declaration of Helsinki. The study protocol was approved by the Institutional Review Board of each participating hospital. Written informed consent was obtained from the patient and healthy subjects.

### Assessment of disease activity

To evaluate changes in disease activity in IBD patients, assessments were made at visit 1 (V1), which represents the visit before initiation of anti-TNF-α treatment, and visit 2 (V2), which represents the visit 3 months after initiation of anti-TNF-α treatment.

In UC patients, disease activity was assessed using the Mayo score. The Mayo score was calculated according to the bowel frequency, rectal bleeding, endoscopic findings, and physician assessment, and each item was scored from 0–3 and summed to a total of 12^53,54^. Using this scale, UC was defined as mild (3–5 points), moderate (6–10 points), and severe (11–12 points)^55^. CD disease activity was assessed using Crohn’s Disease Activity Index (CDAI) and was divided into asymptomatic remission (CDAI < 150), mild-to-moderate CD (CDAI 150–220), moderate-to-severe CD (CDAI 220–450), and severe-fulminant disease (CDAI > 450)^56^.

The response to treatment was defined as clinical and endoscopic improvement, and was measured based on the activity index^57^. In patients with UC, clinical response was defined as a decrease from baseline of ≥ 30% and ≥ 3 points in the Mayo score, along with either a rectal bleeding subscore of 0 or 1 or a decrease from baseline of ≥ 1 in the rectal bleeding subscore, or a reduction by ≥ 2 points and 25% in the partial Mayo score compared with baseline^58^. In CD patients, the response to treatment was defined as a reduction in CDAI of ≥ 70^59^. In addition, in the patients with UC, clinical remission was defined as a Mayo score of ≤ 2, along with not having > 1 point in any individual subscore ^60^. In addition, for CD patients, clinical remission was defined as a CDAI < 150^56^.

To investigate the influence of disease activity on the microbiome at the phylum level, disease activity at V1 in patients with IBD was classified as being in remission, mild-moderate IBD, or severe IBD. In addition, we investigated laboratory results assessed in V1 and V2, including erythrocyte sedimentation rate, C-reactive protein levels, and hematocrit levels. Fecal calprotectin levels were also measured at V1 and V2 in IBD patients, and at baseline, as a control, in healthy individuals.

### Sample collection and analysis methods

The healthy controls provided stool, saliva, serum, and urine samples at baseline, and IBD patients provided these four sample types at V1 and V2 (i.e., before and after anti-TNF-α treatment). Saliva was collected in a 15-cc falcon tube with 5 mL of clear saliva without food intake for at least 1 h, and 2 g of feces was collected in a stool sterilized container. Fecal calprotectin was measured in a single frozen stool sample from all subjects by using Calprotectin Bühlmann ELISA (Bühlmann Laboratories AG, Schönenbuch, Switzerland). Experimental samples were assayed with the standards and controls included with the kit according to the manufacturer’s instructions. For urine samples, 30 mLs were collected, and 5 mLs of blood were collected in serum-separating tubes.

#### Next-generation sequencing (NGS) analysis

DNA extraction was performed by using FastDNA Spin Kit for Soil (MP Biomedicals, Irvine, California, USA) on stool and saliva samples. Polymerase chain reaction (PCR) was then performed to amplify template out of the DNA samples by using V3-V4 region primers with overhang adapters attached, which were 16S_V3_F (5′- TCG TCG GCA GCG TCA GAT GTG TAT AAG AGA CAG CCT ACG GGN GGC WGC AG-3′) and 16S_V4_R (5′-GTC TCG TGG GCT CGG AGA TGT GTA TAA GAG ACA GGA CTA CHV GGG TAT CTA ATC C-3′). After attaching Nextera® XT Index Kit V2, an Illumina adapter primer, sequencing was performed using an Illumina V3 600 cycle cartridge and Illlumina MiSeq equipment (San Diego, California, USA).

#### Extracellular vesicle (EV) analysis

Nanovesicles were separated from the samples through ultracentrifugation, gDNA was extracted, then 16 s rRNA sequencing was performed using Illumina MiSeq (Illumina, USA). Through this process, the gut microbiota was classified, and the correlation between the clinical characteristics and the rRNA abundance derived from a specific microorganism was made. EV analysis was performed on each sample of stool, saliva, serum, and urine, and was conducted by *MD healthcare,* Seoul, Korea.

Bacterial EVs were boiled using a heat block for 40 min at 100 °C and then the remaining particles and waste were removed by centrifugation at 13,000 rpm for 30 min at 4 °C. The DNA was extracted from supernatants using a DNeasy PowerSoil kit (QIAGEN, Germany). The DNA of bacterial EVs in each sample was quantified by QIAxpert (QIAGEN, Germany). V3-V4 regions of the 16 S rDNA gene was amplified with primers; 16S_V3_F (5′-TCGTCGGCAGCGTCAGATGTGTATAAGAGACAGCCTACGGGNGGCWGCAG-3′) and 16S_V4_R(5′-GTCTCGTGGGCTCGGAGATGTGTATAAGAGACAGGACTACHVGGGTATCTAATCC-3′). The library preparation was performed using PCR products and each amplicon was sequenced by MiSeq.

Paired-end reads that matched the adapter sequences were trimmed by Cutadapt version 1.1.6^61^. The resulting FASTQ files containing paired-end reads were merged with CASPER, and then quality filtered with Phred (Q) score-based criteria described by Bokulich^62,63^. Any reads that were shorter than 350 bp or longer than 550 bp after merging, were also discarded. To identify the chimeric sequences, a reference-based chimera detection step was conducted with VSEARCH against the SILVA gold database^64,65^. Next, the sequence reads were clustered into Operational Taxonomic Units (OTUs) using VSEARCH with a closed-reference clustering algorithm under a threshold of 97% sequence similarity against EzBioCloud database (http://doi.org/10.1099/ijsem.0.001755). The representative sequences of the OTUs were finally classified using SILVA 132 database with UCLUST (parallel_assign_taxonomy_uclust.py script on QIIME version 1.9.1) under default parameters^66^.

### Statistical analysis

The baseline characteristics of the control and IBD patient groups were expressed as medians (interquartile range [IQR]) or number of patients (%). The independent Student’s t-test (or Mann–Whitney test) was used to compare continuous variables and the χ^2^ test (or Fisher’s exact test) was used to compare categorical variables, as appropriate. Data were analyzed using SPSS software (version 25.0; IBM Corp., Armonk, NY, USA). *P*-values of < 0.05 were considered statistically significant.

For microbiota analysis in bulk sample NGS analysis, primers were trimmed by using the ChunLab program (ChunLab, Inc., Seoul, Korea), applying a similarity cut-off of 0.8. The EzBioCloud database (https://www.ezbiocloud.net/)67 was used for taxonomic assignment by using BLAST 2.2.22, and pairwise alignments were generated to calculate similarity. The Wilcoxon rank-sum test was used to test the difference between groups in the number of OTUs, Shannon index, and relative abundances of specific taxa. *P* < 0.05 and false discovery rate–adjusted *p*-values < 0.1 were considered significant. Linear discriminant analysis effect size analysis was used to identify significantly different taxa between the groups^22^.

In the bulk sample NGS analysis, α-diversity (ACE, Chao1, Jackknife, Shannon’s diversity index, NP Shannon, and Simpson indexes) and β-diversity metrics (Bray–Curtis dissimilarity and generated principal coordinates analysis [PCoA]) plot were computed using multidimensional scaling for each of the β-diversity metrics by using EzBioCloud MTP pipeline (http://doi.org/10.1099/ijsem.0.001755).

In EV sample analysis, the rarefaction curve of Chao1 was used for α-diversity using multiple_rarefaction.py and alpha_diversity.py QIIME package (version 1.9.1). The Phyloseq package was used for alpha diversity, and bray–curtis dissimilarity metrics from vegan package for beta diversity. Differences between the relative abundance of microbiota were calculated by the Kruskal–Wallis test and Wilcoxon test. *P*-values of < 0.05 were considered statistically significant. Grouped comparisons of data were conducted with R software (ver 3.6.3; R Core Team 2020, Vienna, Austria) and GraphPad Prism 7.0 (GraphPad Software, Inc., San Diego, CA).

## Supplementary Information

Supplementary Information.

## Data Availability

The data underlying this article will be shared on reasonable request to the corresponding author.
