# Supplementary material for: Microbial changes in stool, saliva, serum, and urine before and after anti-TNF-α therapy in patients with inflammatory bowel diseases
Source: Sci Rep. 2022 Apr 15;12:6359. doi: 10.1038/s41598-022-10450-2 (PMC9012770; doi:10.1038/s41598-022-10450-2)

**Scientific Reports**

**Supplementary Data**

**Microbial changes in stool, saliva, serum, and urine before and after anti-TNF-α therapy in patients with inflammatory bowel diseases**

Yong Eun Park^1,2^, Hye Su Moon^3^, Dongeun Yong^3^, Hochan Seo^4^, Jinho Yang^4^, Tae-Seop Shin^4^, Yoon-Keun Kim^4^, Jin Ran Kim^5^, Yoo Na Lee^5^, Young-Ho Kim^6^, Joo Sung Kim^7^, and Jae Hee Cheon^1,8,*^

^1^Department of Internal Medicine, Yonsei University College of Medicine, Seoul 03722, Republic of Korea

^2^Division of Gastroenterology, Department of Internal Medicine, Inje University College of Medicine, Haeundae Paik Hospital, Busan, Republic of Korea

^3^Department of Laboratory Medicine and Research Institute of Bacterial Resistance, Yonsei University College of Medicine, Seoul 03722, Republic of Korea

^4^MD Healthcare Inc, Seoul, Republic of Korea

^5^Eisai Korea Inc., Seoul, Republic of Korea

^6^Samsung Medical Center, Sungkyunkwan University School of Medicine, Seoul, Korea ^7^Department of Internal Medicine, Seoul National University College of Medicine, Seoul 03080, Republic of Korea

^8^Institute of Gastroenterology, Yonsei University College of Medicine, Seoul 03722, Republic of Korea

**Corresponding author**

Jae Hee Cheon, MD, PhD

Department of Internal Medicine, Yonsei University College of Medicine

50-1 Yonsei-ro, Seodaemun-gu, Seoul 03722, Korea

E-mail: [GENIUSHEE@yuhs.ac](file:///C:\Users\Boats\Desktop\SWPS\Eisai%20Micro%20changes%20TNF\geniushee@yuhs.ac)

Tel.: +82-2-2228-1990

Fax: +82-2-393-6884

**Supplemental Table S1.** Baseline characteristics of study patients. The independent Student’s t-test (or Mann-Whitney test) was used for continuous variables and the χ^2^ test (or Fisher’s exact test) was used for categorical variables.

| **Variables** | **Crohn’s disease**  **(*n =* 10)** | **Ulcerative colitis (*n =* 9)** | ***p-*value (CV vs UC)** |
| --- | --- | --- | --- |
| Male, n (%) | 7 (70.0) | 6 (66.7) | 0.876 |
| Age in years, median (IQR) | 31 (23-40) | 52 (30-64) | 0.021 |
| ***Underlying diseases, n* (%)** |  |  |  |
| Hypertension | 1 (10.0) | 1 (11.1) | 0.937 |
| Hematologic disorder | 0 (0.0) | 1 (11.1) | 0.279 |
| Eye disorder (vitreous floater) | 0 (0) | 1 (11.1) | 0.279 |
| Hemorrhoid, anorectal disorder | 1 (10.0) | 0 (0) | 0.330 |
| Subdural hemorrhage | 1 (10.0) | 0 (0) | 0.330 |
| Viral hepatitis C | 1 (10.0) | 0 (0) | 0.330 |
| ***Medications, n* (%)** |  |  |  |
| 5-aminosalicylic acid | 9 (90.0) | 9 (100.0) | 0.330 |
| Immunomodulators | 9 (90.0) | 3 (33.3) | 0.011 |
| Steroids | 5 (50.0) | 5 (55.6) | 0.809 |
| Others |  |  | 0.403 |
| Iron supplement | 1 (10.0) | 0 (0) |  |
| Pain killer | 1 (10.0) | 1 (11.1) |  |
| Anti-spasmodic | 0 (0) | 1 (11.1) |  |
| Folic acid | 1 (10.0) | 0 (0) |  |
| ***Anti-TNF-α agent initiated after visit 1, n* (%)** | | | 0.137 |
| Infliximab | 7 (70.0) | 4 (44.4) |  |
| Adalimumab | 3 (30.0) | 2 (22.2) |  |
| Golimumab | 0 (0) | 3 (33.3) |  |

*IQR* interquartile range, *TNF* tumor necrosis factor

**Supplemental Table S2.** *Firmicutes/Bacteroidetes* ratio in stool, saliva, serum, and urine samples of the control group at baseline, and the inflammatory bowel disease group before (V1) and after (V2) anti-TNF-α treatment grouped by their clinical response.

| **Variables** | ***Firmicutes/Bacteroidetes* ratio ± standard deviation** | | | |
| --- | --- | --- | --- | --- |
|  | **Stool** | **Saliva** | **Serum** | **Urine** |
| **Control**  ***V1***  Responder | 8.78 ± 15.78  624.72 ± 2035.31  265.07 ± 762.87 | 3.81 ± 4.23  3.31 ± 2.53  2.35 ± 0.87 | 2.00 ± 1.68  5.32 ± 6.69  8.05 ± 8.07 | 26.06 ± 74.36  6.89 ± 6.37  4.32 ± 4.62 |
| Non-responder | 1 029.32 ± 2 906.89 | 4.27 ± 3.29 | 1.91 ± 0.97 | 9.75 ± 7.06 |
| *p*-Value (response vs non-response; t-test)  ***V2***  Responder | 0.458  21.25 ± 51.23  31.37 ± 63.77 | 0.125  110.45 ± 432.06  4.86 ± 3.17 | 0.040  6.30 ± 7.06  6.51 ± 8.02 | 0.061  16.59 ± 41.77  22.43 ± 55.59 |
| Non-responder | 4.38 ± 2.30 | 261.30 ± 672.80 | 6.05 ± 6.42 | 9.07 ± 11.20 |
| *p*-Value (response vs non-response; t-test) | 0.324 | 0.352 | 0.906 | 0.544 |
| *p*-Value (control vs V1 vs V2; Kruskal-Wallis) | 0.300 | 0.251 | 0.058 | 0.569 |

**Supplemental Figure S1.** Diversity in the control and the inflammatory bowel disease (IBD) groups before (V1) and 3 months after anti-TNF-α treatment (V2). α-Diversity using next-generation sequencing in: (A) stool (Jackknife and NP Shannon); and (B) saliva (Jackknife, NP Shannon, ACE, Chao1, Shannon, and Simpson index). (C) β-diversity analysis in saliva (Principal coordinate analysis scatter plot). Differences between the relative abundance of microbiota were calculated by the Kruskal–Wallis test and Wilcoxon test.

**
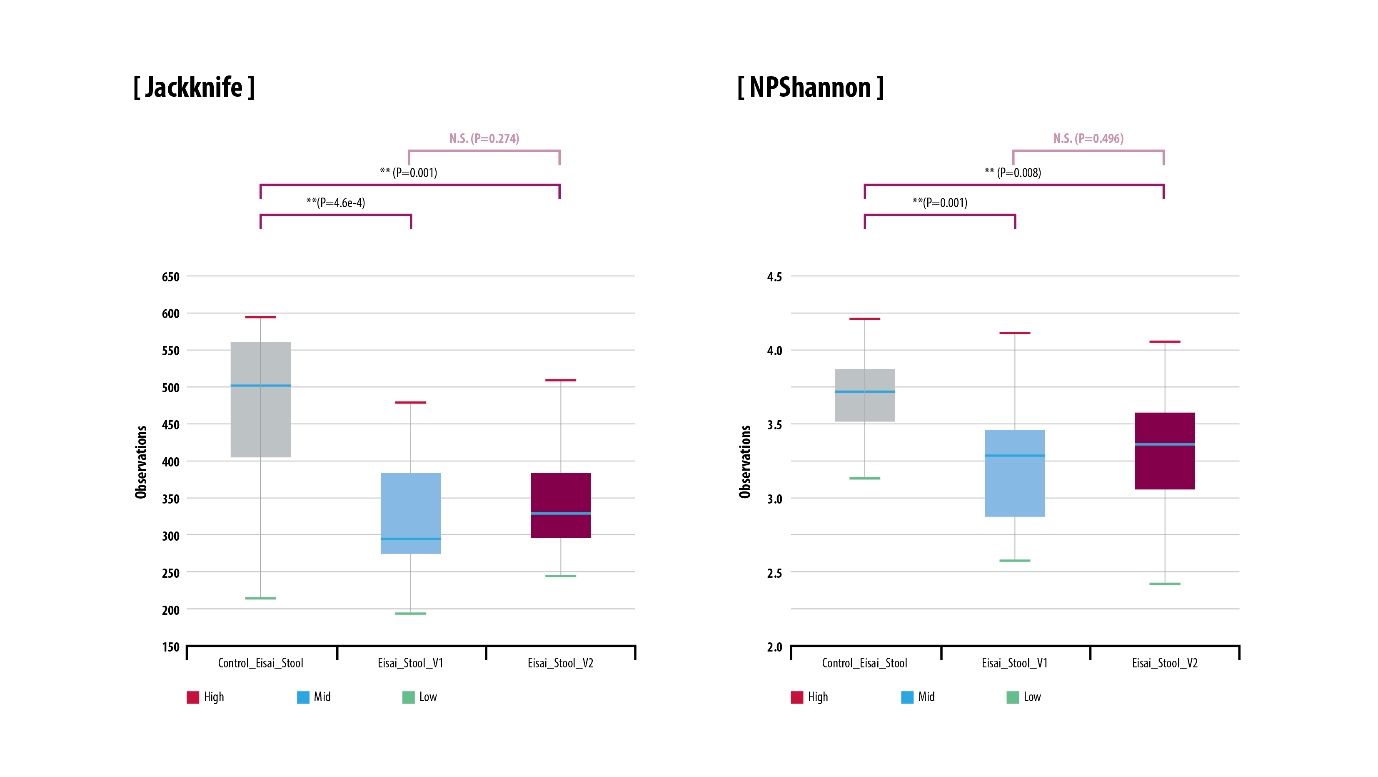

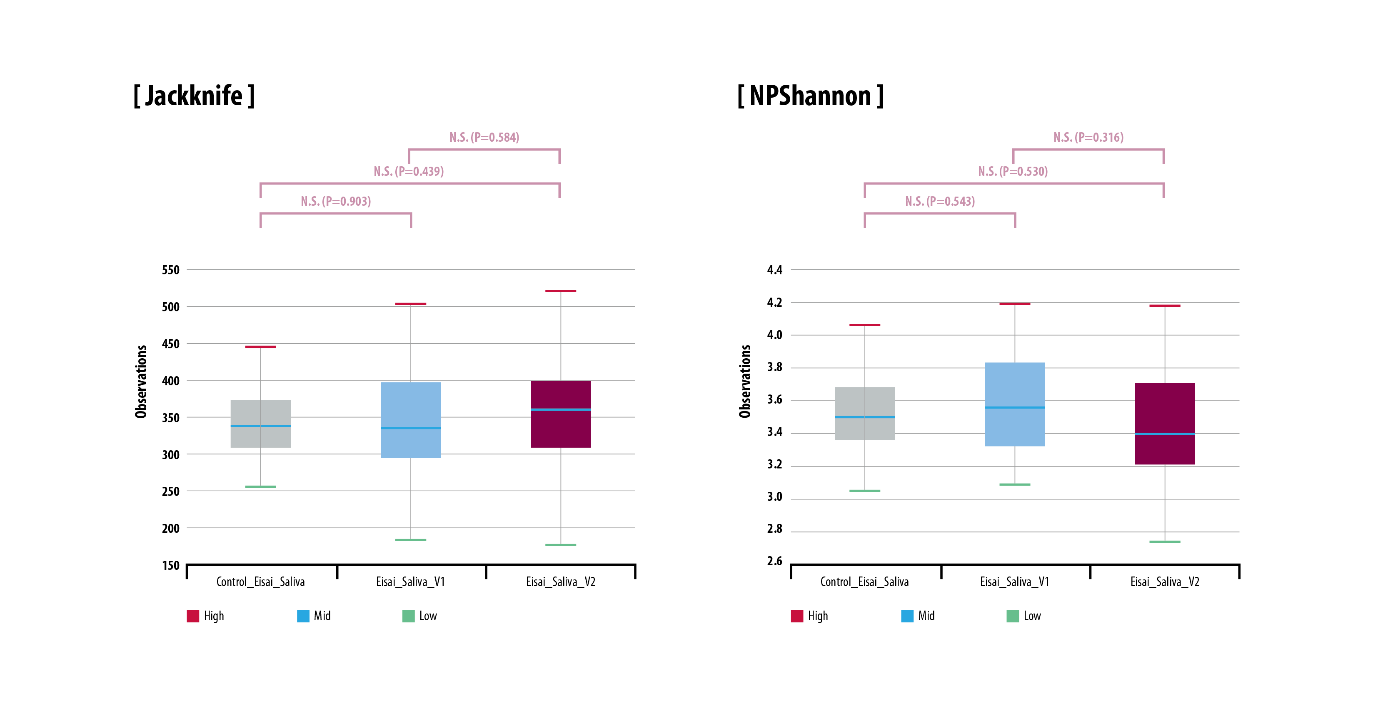
**

**(B)**

**(A)**

**
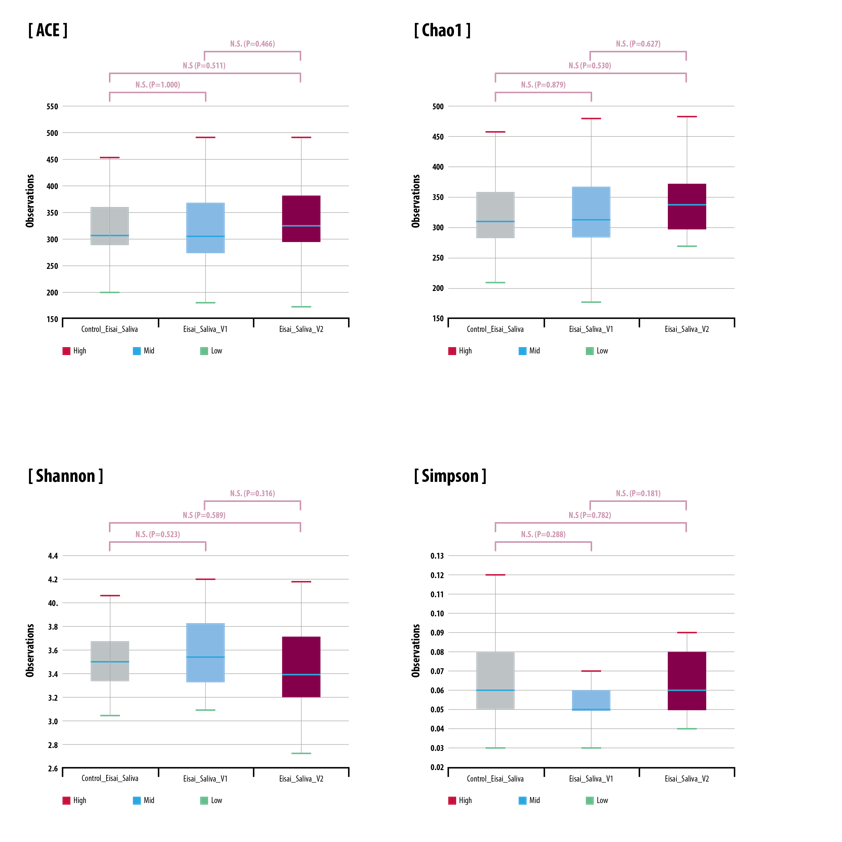
**

**
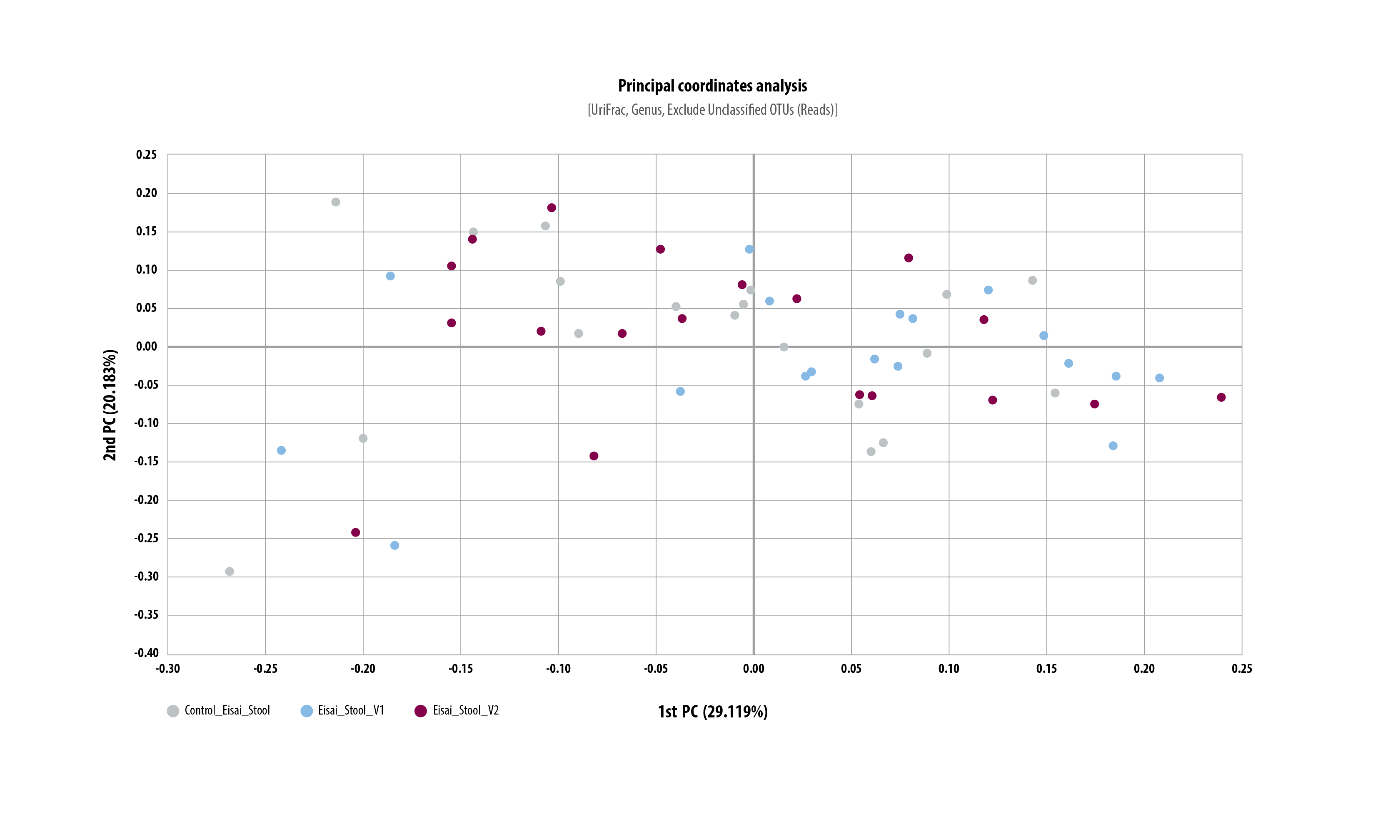
**

**(C)**

**Supplemental Figure S2.** α-Diversity using extracellular vesicle analysis in the control group at baseline, and the inflammatory bowel disease (IBD) group before (V1) and after (V2) anti-TNF-α treatment in: (A) stool; (B) serum; (C) saliva; and (D) urine [rarefied Chao 1 plot] (*p* > 0.05)
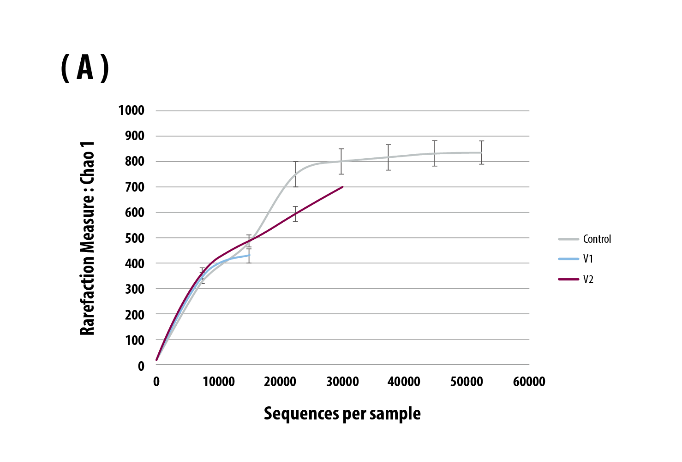

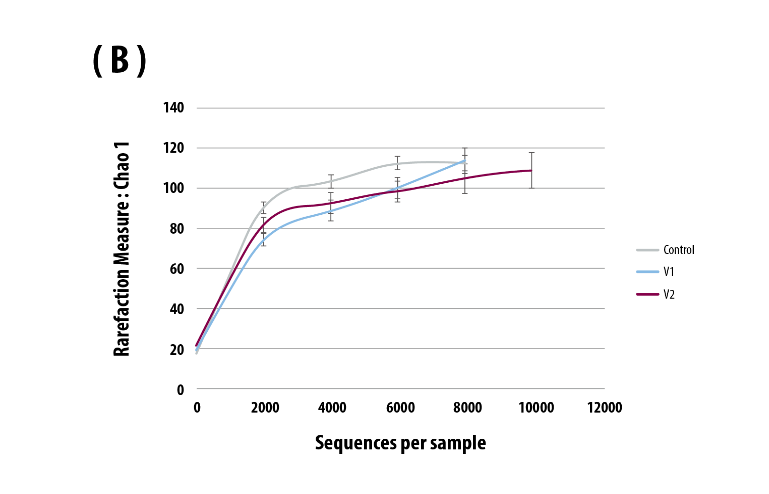
**
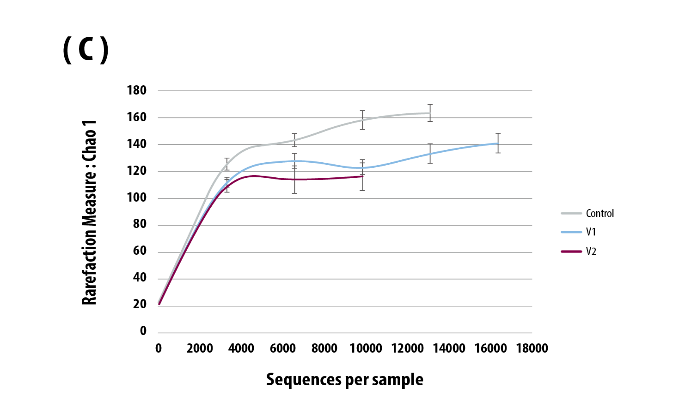
**

**
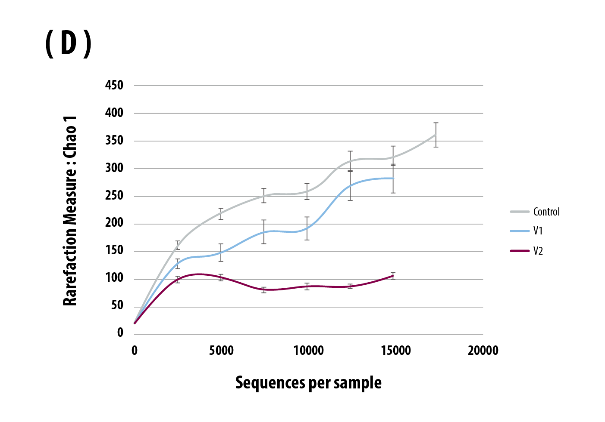
**

**Supplemental Figure S3.** β-diversity of stool in the control group at baseline, and the inflammatory bowel disease (IBD) group before (V1) and 3 months after anti-TNF-α treatment (V2) in extracellular vesicle analysis according to: (A) Phylum; (B) Class; (C) Order; (D) Family; (E) Genus; and (F) Species.


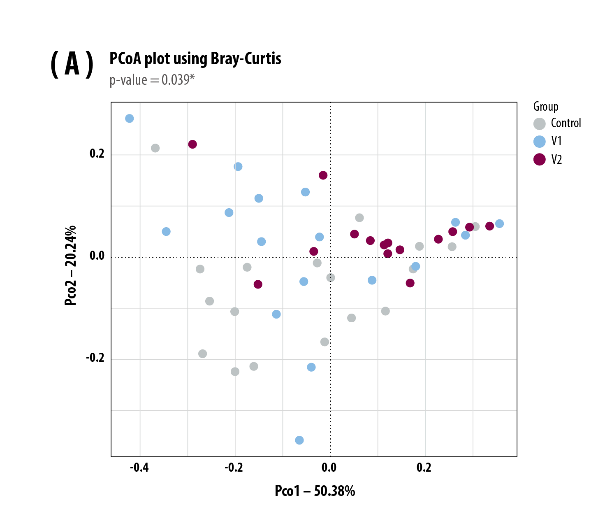

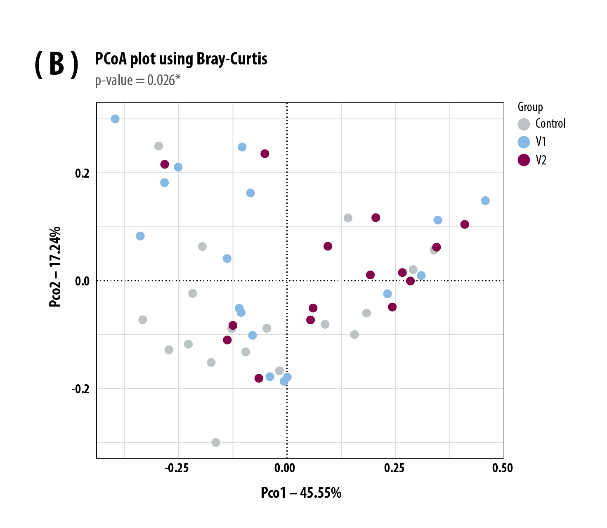


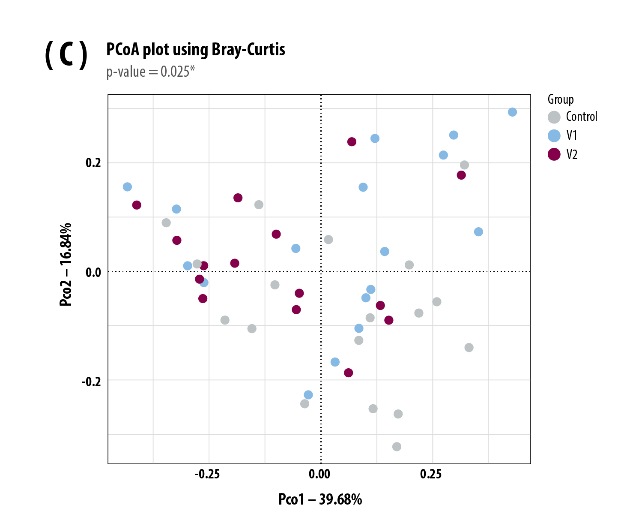

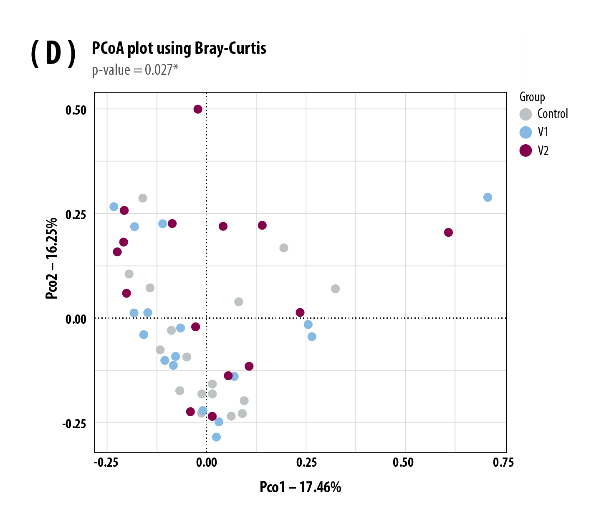


**
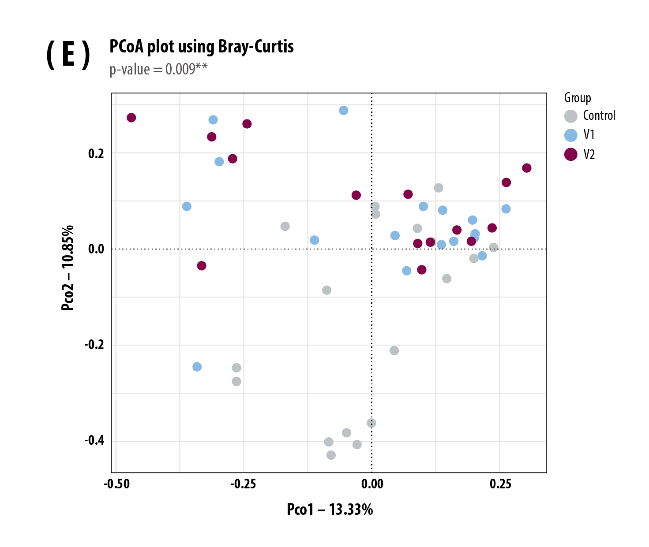

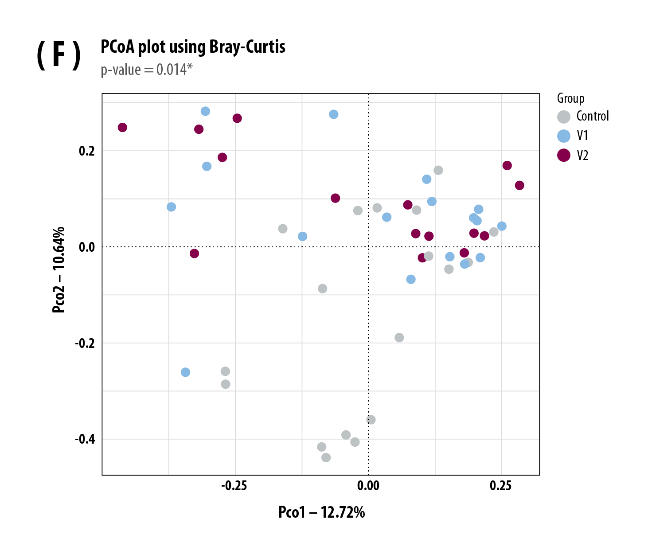
**

**Supplemental Figure S4.** Differences between the control and inflammatory bowel disease (IBD) groups before (V1) and 3 months after anti-TNF-α treatment (V2). Next-generation sequencing analysis of microbial composition in: (A) stool; and (B) saliva. Extracellular vesicle (EV) analysis of the mean relative abundance of the microbiome in: (C) stool, phylum and class level; and (D) saliva, phylum and family levels. Kruskal–Wallis and Wilcoxon tests were used. Bars above columns indicate standard deviation. * *p* < 0.05, ** *p* < 0.01


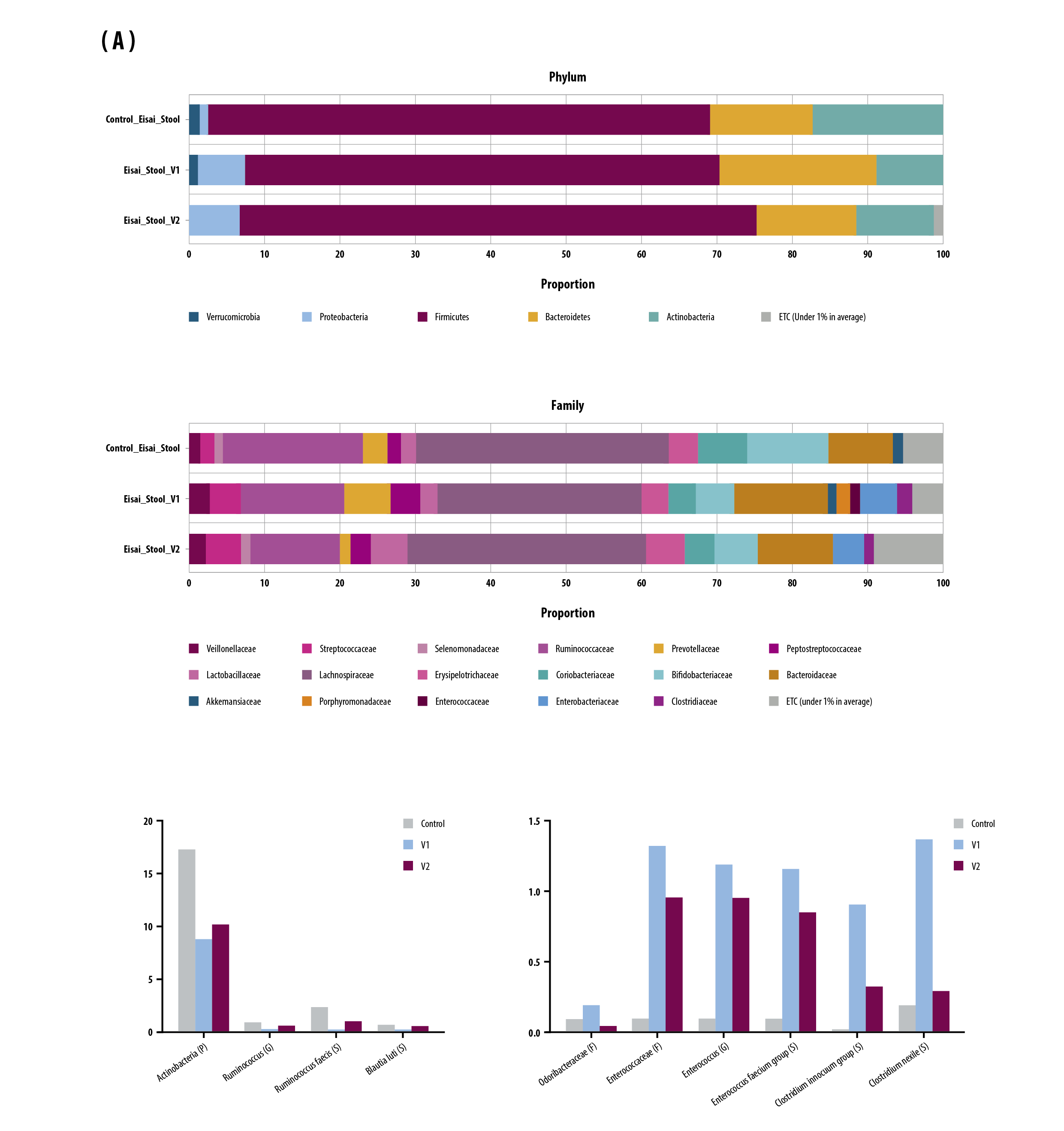

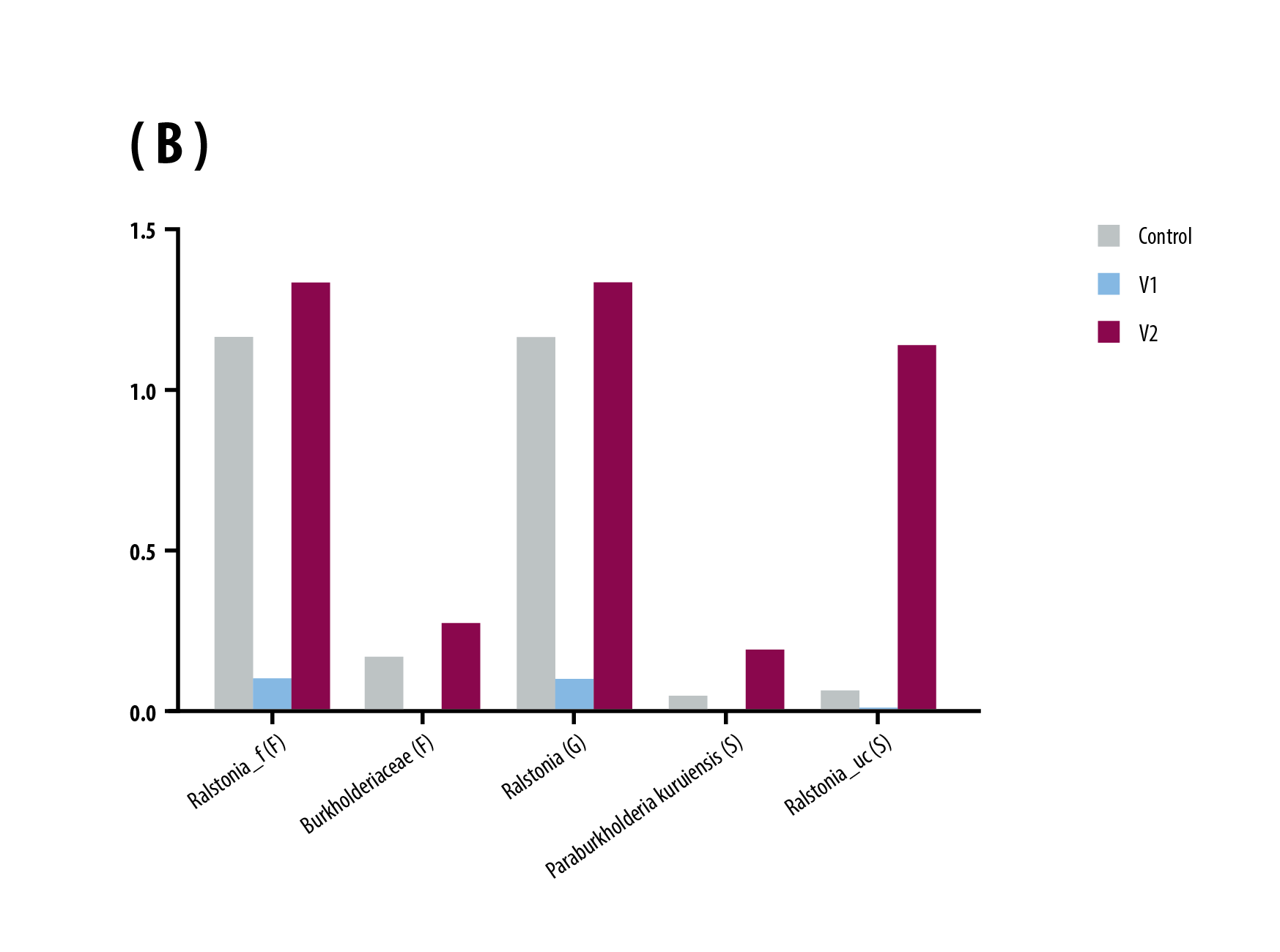

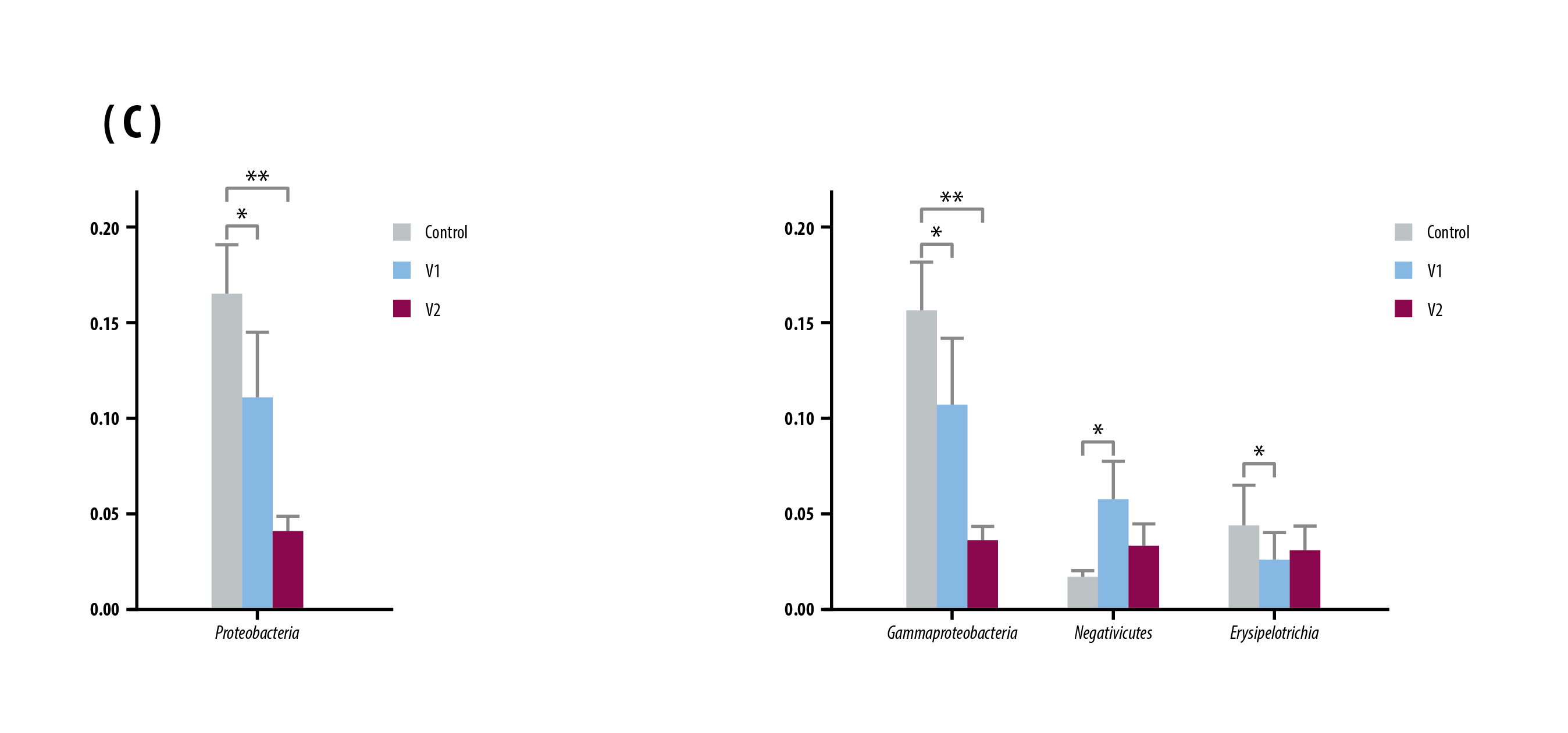


**
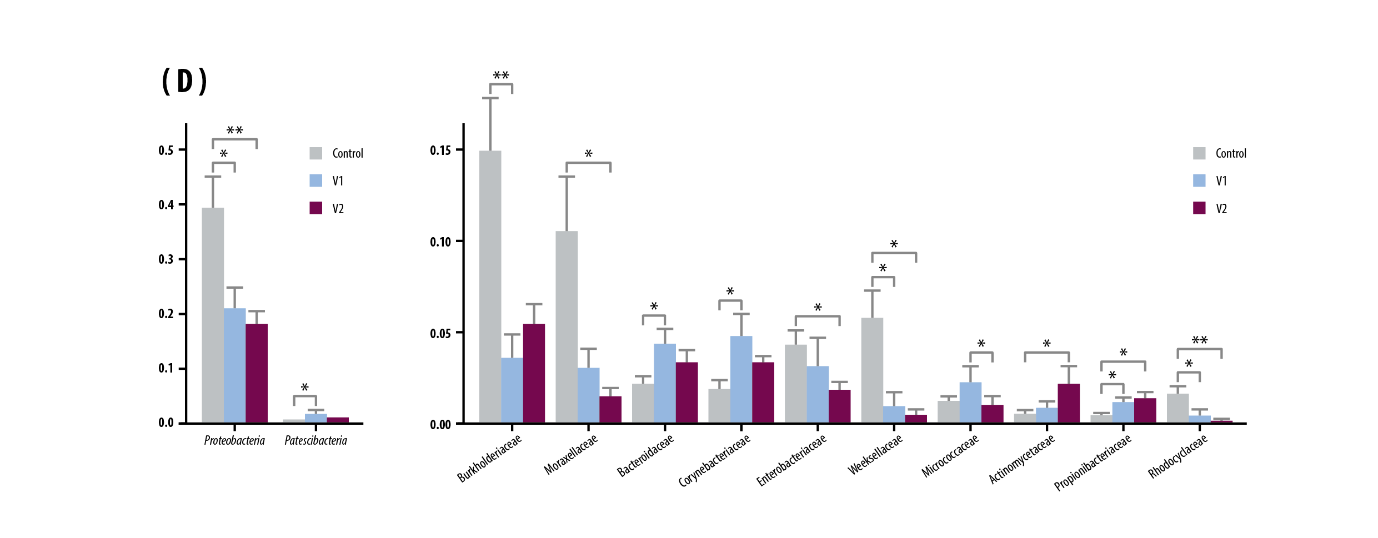
**

**Supplemental Figure S5.** Microbials of the control group and the inflammatory bowel disease (IBD) group before anti-TNF-α treatment (V1) between patients with and without remission. Mean relative abundance in: (A) stool, microbials abundant in the remission group; (B) serum, microbials abundant in the non-remission group. Kruskal–Wallis and Wilcoxon tests were used. Bars above columns indicate standard deviation. * *p* < 0.05.


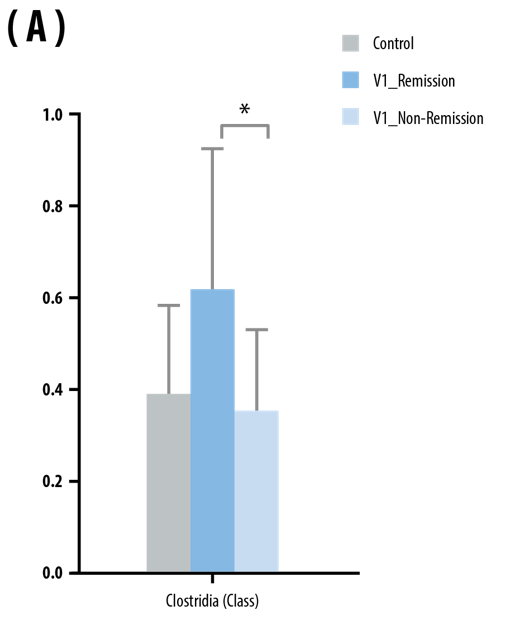


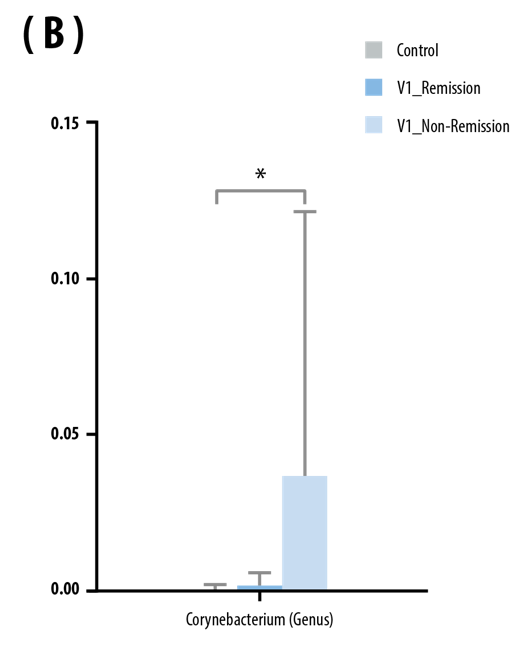


**Supplemental Figure S6.** Microbiome (extracellular vesicles analysis) in the inflammatory bowel disease (IBD) group with significant differences in in all four samples (stool, saliva, serum, urine) before anti-TNF-α treatment (V1). Mean relative abundance of *Acidovorax caen*i at V1 in: (A) patients with and without response; and (B) patients with and without remission. Kruskal–Wallis and Wilcoxon tests were used. Bars above columns indicate standard deviation. All *p* < 0.05.


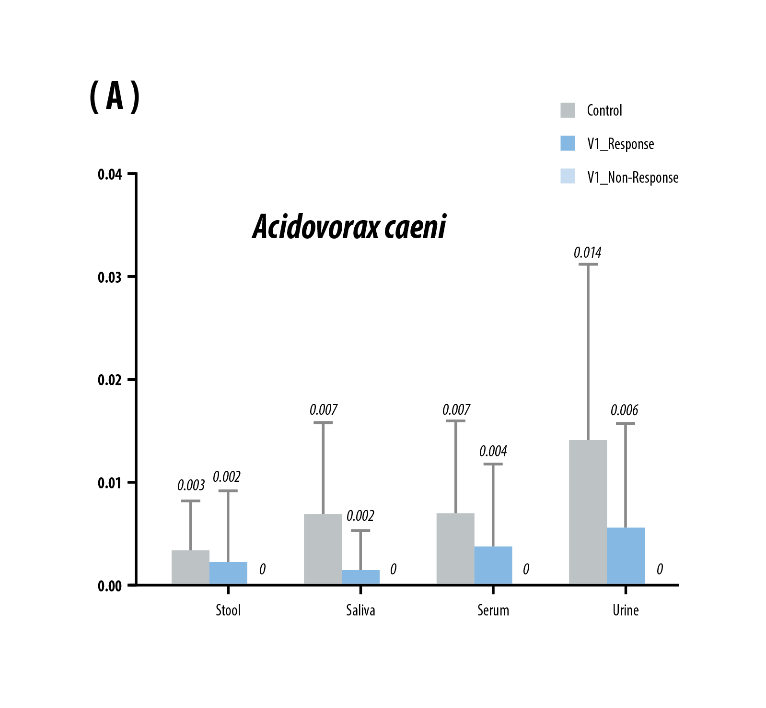


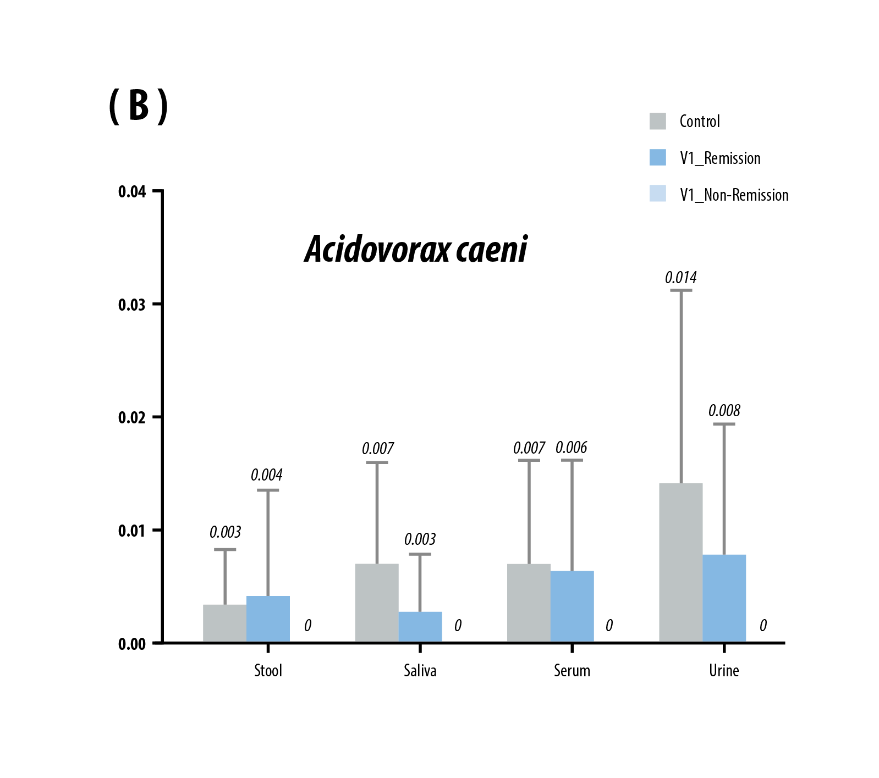

Supplement: Supplementary file 1 — Supplementary Information. [file 41598_2022_10450_MOESM1_ESM.docx]
